# Insufficient iodine nutrition status and the risk of pre-eclampsia: a systemic review and meta-analysis

**DOI:** 10.1136/bmjopen-2020-043505

**Published:** 2021-02-10

**Authors:** Charles Bitamazire Businge, Anthony Usenbo, Benjamin Longo-Mbenza, AP Kengne

**Affiliations:** 1 Department of Obstetrics and Gynaecology, Faculty of Health Sciences, Walter Sisulu University, Mthatha, South Africa; 2 Department of Medicine, Faculty of Health Sciences, University of Cape Town, Cape Town, South Africa; 3 Department of Anaesthesiolgy, Nelson Mandela Academic Hospital, Mthatha, South Africa; 4 Faculty of Medicine, University of Kinshasa and LOMO University of Research, Kinshasa, Democratic Republic of Congo; 5 Non-Communicable Disease Research Unit, South African Medical Research Council, Cape Town, South Africa

**Keywords:** maternal medicine, epidemiology, nutrition & dietetics

## Abstract

**Background:**

Although subclinical hypothyroidism in pregnancy is one of the established risk factors for pre-eclampsia, the link between iodine deficiency, the main cause of hypothyroidism, and pre-eclampsia remains uncertain. We conducted a systematic review to determine the iodine nutrition status of pregnant women with and without pre-eclampsia and the risk of pre-eclampsia due to iodine deficiency.

**Methods:**

MEDLINE, EMBASE, Google Scholar, Scopus and Africa-Wide Information were searched up to 30th June 2020. Random-effect model meta-analysis was used to pool mean difference in urinary iodine concentration (UIC) between pre-eclamptic and normotensive controls and pool ORs and incidence rates of pre-eclampsia among women with UIC <150 µg/L.

**Results:**

Five eligible studies were included in the meta-analysis. There was a significant difference in the pooled mean UIC of 254 pre-eclamptic women and 210 normotensive controls enrolled in three eligible case–control studies (mean UIC 164.4 µg/L (95% CI 45.1 to 283.6, p<0.01, I^2^ >50)). The overall proportions of pre-eclampsia among women with UIC <150 µg/L and UIC >150 µg/L in two cross-sectional studies were 203/214 and 67/247, respectively, with a pooled OR of 0.01 (95% CI 0 to 4.23, p=0.14, I^2^ >50) for pre-eclampsia among women with UIC >150 µg/L. The overall incidence of pre-eclampsia among women with UIC <150 µg/L and UIC >150 µg/L in two cohort studies was 6/1411 and 3/2478, respectively, with a pooled risk ratio of 2.85 (95% CI 0.42 to 20.05, p=0.09, I^2^ <25).

**Conclusion:**

Although pre-eclamptic women seem to have lower UIC than normotensive pregnant women, the available data are insufficient to provide a conclusive answer on association of iodine deficiency with pre-eclampsia risk.

**PROSPERO registration number:**

CRD42018099427.

**Strengths and limitations of this study**

- The current study is among the first systematic reviews that have ascertained the relationship between insufficient iodine nutrition status and pre-eclampsia.
- This review has however been limited by the small number of eligible studies coupled with small sample sizes.
- The varied study designs coupled with a considerable degree of heterogeneity precluded the pooling of all the results.

## Introduction

Subclinical hypothyroidism is a risk factor for pre-eclampsia, which is a prominent cause of maternal and perinatal morbidity and mortality.^1–3^ Iodine deficiency, which is exacerbated by pregnancy-related physiological changes, is a leading cause of hypothyroidism.^4 5^ Hence, among women within the reproductive age bracket, insufficient nutrition status prior to the onset of pregnancy, which potentially worsens during the course of pregnancy, could increase the risk of pre-eclampsia like is the case for fetal neurological complications, particularly in endemic iodine deficiency settings.^6 7^

Over two billion people live in areas with iodine insufficiency.^8^ Iodine deficiency is on the rise in areas originally thought to be iodine sufficient, despite concerted worldwide efforts to promote iodine fortification. This is partly attributed to high concentration of perchlorate and thiocyanate in water sources and the diet, which impairs the uptake of iodine by the thyroid gland, particularly among individuals with thyroid stimulating hormone (TSH)-related thyroid stimulation secondary to iodine deficiency, and to ineffective implementation and monitoring of dairy-based and bread-based iodine supplementation strategies.^9–12^

In this systematic review and meta-analysis, we sought to establish if there is a difference in the urinary iodine concentration (UIC) of pregnant women with and without pre-eclampsia and whether pregnant women with insufficient iodine nutrition status are at increased risk of pre-eclampsia.

The study is reported according to the Preferred Reporting Items for Systematic reviews and Meta-Analysis guidelines^13^ and was based on a protocol that was registered with the International Prospective Register of Systematic Reviews.

## Methods

### Eligibility criteria

#### Inclusion criteria

The selection of studies for inclusion was guided by the Population, Intervention/Exposure, Comparison and Outcome protocol. The target population was pregnant women, and the exposure was insufficient iodine nutrition status before pregnancy for cohort studies and insufficient iodine nutrition status during pregnancy for case–control studies. The iodine nutrition status was defined according to the WHO/International Council for Control of Iodine Deficiency Disorders classification of iodine intake using median UIC.^14 15^ For pregnant women, a UIC <150, 150–249, 250–499 and >500 µg/L are considered an estimate of insufficient, adequate, more than adequate and excessive iodine nutritional status, respectively.^15^ The comparators were study participants with sufficient iodine nutrition status (UIC ≥150 µg/L) during pregnancy.^14 15^ The outcomes were the prevalence and incidence rates of pre-eclampsia among women with and without adequate iodine nutrition status from which the ORs for case–control and risk ratios for cohort studies were determined.

Pre-eclampsia was defined as new-onset hypertension after 20 weeks of amenorrhoea characterised by elevated systolic blood pressure (SBP >140 mm Hg) and/or diastolic blood pressure (DBP >90 mm Hg), based on two measurements 4 hours apart, or SBP >160 mm Hg and/or DBP >110 mm Hg from a single measurement. Elevated blood pressure (BP) had to be accompanied by at least one of the following: proteinuria in 24 hour urine ≥300 mg or protein/creatinine ratio ≥0.3 mg/mg or urine protein measured by dipstick ≥2+, thrombocytopenia (platelet count less than 150 x 10^9^L), kidney insufficiency (serum creatinine levels above 90 μmol/L), decreased liver function (AST and ALT twice higher than the upper limit of the reference interval), compromised lung function or pulmonary oedema, visual or other symptoms and signs of impaired cerebral function.^16^ There may be considerable heterogeneity if pre-eclampsia has been variably defined in different studies that are eligible for inclusion in the current systematic review.

### Exclusion criteria

Studies were excluded if they lacked means, medians, ORs, incidence and prevalence rate data to compute them even after repeated unsuccessful attempts to contact the authors via email for relevant information. Letters to editors, reviews, commentaries, editorials and any publication without primary data were also excluded.

### Patient and public involvement

There was no involvement of the public or patients.

### Search strategy and selection criteria

We searched PubMed, Scopus, Web of Science, Academic Search Premier, Africa-Wide Information, CINAHL, Cochrane Library, Google Scholar and Health Source: Nursing/Academic Edition databases for all published studies on iodine deficiency and pre-eclampsia up to 30th June 2020. This search was conducted using a predefined comprehensive and sensitive search strategy (table 1) combining relevant terms and synonyms, which are variably used to denote abnormally high BP in pregnancy and insufficient iodine intake or iodine deficiency as detailed in the published protocol for this review.^17^

**Table 1:** Search strategy for MEDLINE^15^

Population: **pregnant** women with pre-eclampsia		
#1	MeSH terms	Pregnant Women [Mesh] OR Pregnancy [Mesh] OR Pregnancy Trimesters [Mesh]
#2	Free text	Pregnancy OR Pregnant women OR expectant mothers
#3	#1 OR #2	
#4	MeSH terms	Pre-Eclampsia [Mesh] OR Eclampsia [Mesh] OR Hypertension [Mesh]
#5	Free text	Preeclampsia OR Pre-eclampsia OR Eclampsia OR Hypertension OR Hypertensive OR High blood pressure
#6	#4 OR #5	
Exposure: iodine deficiency		
#7	MeSH terms	Iodine [Mesh]
#8	Free text	Iodine
#9	#7 OR #8	
#10	#3 AND #6 AND #9	

MeSH, Medical Subject Headings.

### Study selection and data extraction

**Figure 1:**
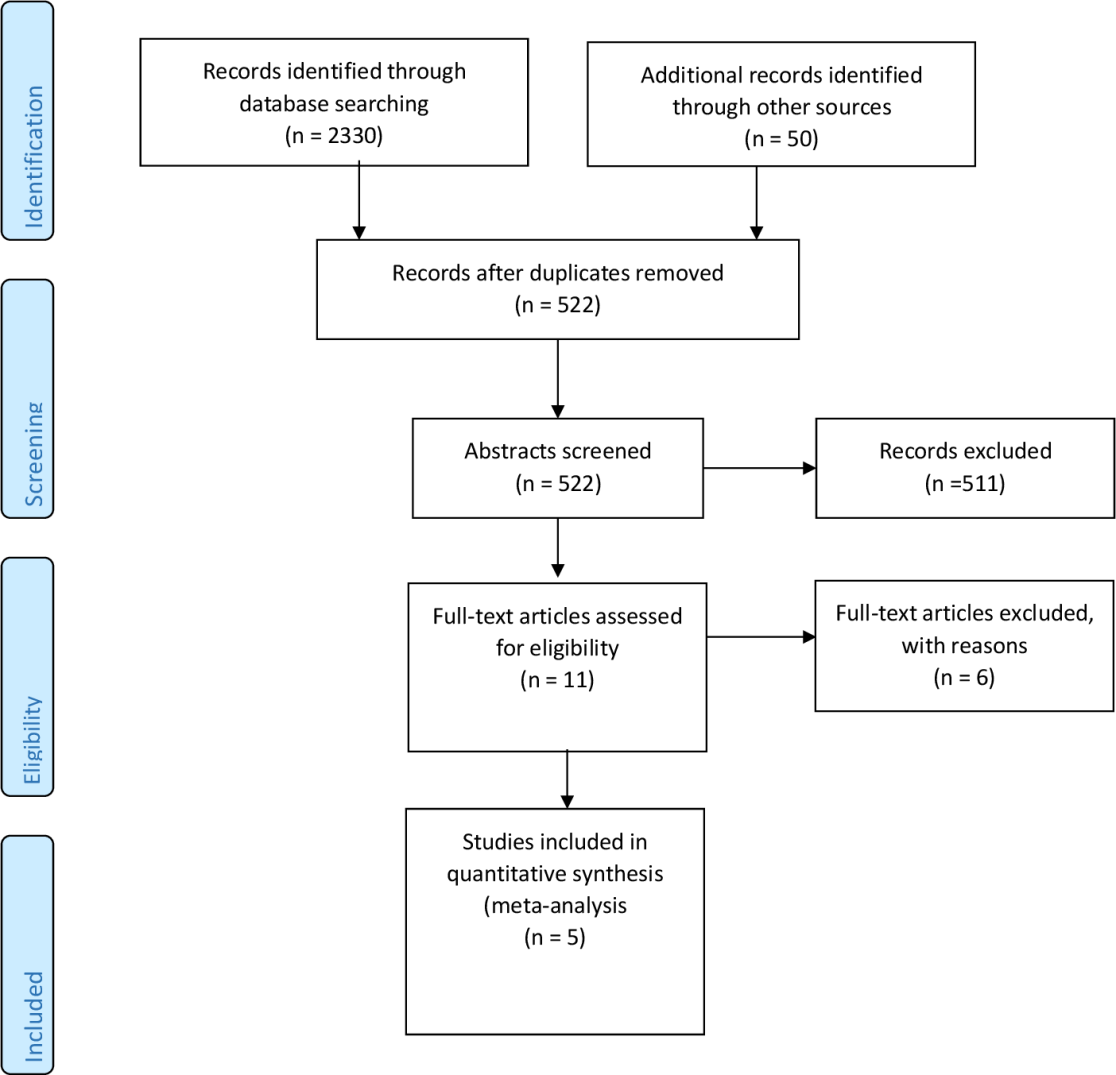
Study selection flow chart.

Two authors (CBB and AU) independently screened the titles and abstracts of identified studies. Citations and abstracts were initially screened and duplicate citations excluded. Titles and abstracts were then screened following inclusion criteria described in the protocol,^17^ after which the full texts of potentially eligible articles were obtained. These full texts were screened using a standardised and pretested form to include eligible studies. Disagreements were resolved by consensus. For each study, one reviewer (CBB) extracted the data, and a second reviewer (AU) checked the accuracy. For the five studies included here, there were no disagreements between the two reviewers. Figure 1 shows the flow chart for the selection process. The following data were extracted from the eligible studies: study characteristics (authors, years, design and study regions), study population (age and sample size), iodine nutrition status of the various study groups and the methods of outcome measurement.

### Quality assessment

Two reviewers (CBB and AU) independently scored the risk of bias and the quality of included studies (table 2A, B) using the Newcastle-Ottawa Scale.^18^ Inter-rater agreement on screening, data abstraction and methodological quality (selection, comparability of groups and ascertainment of exposure/outcome) was assessed using Cohen’s κ coefficient.^19^ The kappa value for inter-rater agreement for quality assessment was 0.694 (p<0.001). Discrepancies were resolved by consensus.

**Table 2:** (A) Risk of bias assessment (reviewer: CBB). (B) Risk of bias assessment (reviewer: AU)

Study	Study type	Selection	Comparability	Outcome/exposure	AHRQ Scale (good/fair/poor)
(A)					
23	Case–control	***	**	***	Good
Cuellar-Rufino *et al*^24^	Case–control	****	**	***	Good
Businge *et al*^25^	Case–control	****	**	**	Good
Yang *et al*^26^	Cohort	****	**	***	Good
27	Cohort	****	**	***	Good
(B)					
Gulaboglu *et al*^23^	Case–control	***	**	***	Good
Cuellar-Rufino *et al*^24^	Case–control	***	**	**	Good
Businge *et al*^25^	Case–control	****	**	***	Good
Yang *et al*^26^	Cohort	****	**	***	Good
27	Cohort	****	**	***	Good

AHQR, Agency for Healthcare Research and Quality.

### Data synthesis, analysis and assessment of heterogeneity

The analysis was performed with the Review Manager (RevMan) Software, V.5.4 (the Nordic Cochrane Centre, the Cochrane Collaboration) and the ‘meta’ and ‘metafor’ packages of the statistical software R (V.4.0.2, the R Foundation for Statistical Computing, Vienna, Austria). For the outcomes of interest (means, prevalence and incidence rates), random-effect model meta-analyses were used to pool estimates across studies with similar design.^20^ The degree of heterogeneity across studies was assessed using the Cochrane Q Statistic and Inconsistency Index (I^2^) (statistics and values ranked as indicating low, I^2^ <25%; moderate, 25%–50%; and high heterogeneity, I^2^ >50%).^21^ The Egger funnel plot was used to check for publication bias.^22^

## Results

### The review process

The process for selecting the relevant studies is summarised in figure 1. In total, 2380 records were identified via database searches. After removing duplicates, we scanned the titles and abstracts of 522 articles, of which 11 full texts were further reviewed. Of these, five articles met criteria for inclusion in the current systematic review.

### Characteristics of included studies

All the five included studies were categorised as having a low risk of bias. Their characteristics are summarised in table 3. Three were institutional-based case–control studies one from the countries Turkey, Mexico and the Democratic Republic of Congo, while two were prospective cohort studies that were from two different provinces (Henan and Liaoning) in China.^23–28^

**Table 3:** Characteristics of included studies

Study	Country	Study design	Study period	Cases (n)	Controls (n)	Comparator	Diagnostic criteria
23	Turkey	Case–control	Not stated	Severe pre-eclampsia (40)	Normotensives (18)	Mean UIC	Sandell-Kolthoff reaction
Cuellar-Rufino *et al*^24^	Mexico	Case–control	Jan–April 2015	Pre-eclampsia (20)	Normotensives (37)	Mean UIC UIC <150 µg/L	Fast colorimetric method
Businge *et al*^25^	Democratic Republic of Congo	Case–control	Jan 2007 to December 2008	Pre-eclampsia (68) and severe pre-eclampsia/eclampsia (182)	Normotensives (150)	Mean UIC UIC <150 µg/L	Sandell-Kolthoff reaction
Yang *et al*^26^	Henan Province, China	Cohort	July to September 2015	Incident pre-eclampsia 1/718 for women with UIC <150 µg/L Incident gestational HT 17/718 for women with UIC <150 µg/L	Incident pre-eclampsia 1/1602 for women with UIC >150 µg/L Incident gestational HT 25/1602 for women with UIC >150 µg/L	UIC <150 µg/L	Fast colorimetric method
27	Liaoning Province, China	Cohort	2012–2014	Incident pre-eclampsia 5/693 for women with UIC <150 µg/L Incident gestational HT 18/693 for women with UIC <150 µg/L	Incident pre-eclampsia 2/876 for women with UIC >150 µg/L Incident gestational HT 25/876 for women with UIC >150 µg/L	UIC <150 µg/L	Sandell-Kolthoff reaction

HT, hypertension; UIC, urinary iodine concentration.

### Meta-analysis

#### Mean difference in UIC of pre-eclamptic and normotensive women

**Figure 2:**
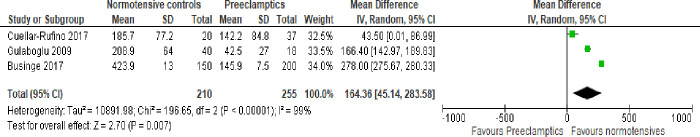
Forest plot showing the mean difference in urinary iodine concentration of normotensive and pre-eclamptic mothers.

Three studies reported the mean UIC of pre-eclamptic and normotensive pregnant women.^23–25^ Overall, there was a significant and positive mean difference in UIC and standardised mean UIC of normotensive pregnant women and pre-eclamptic women, with substantial heterogeneity across studies (figure 2).

### The risk of pre-eclampsia among women with UIC <150 µg/L

**Figure 3:**
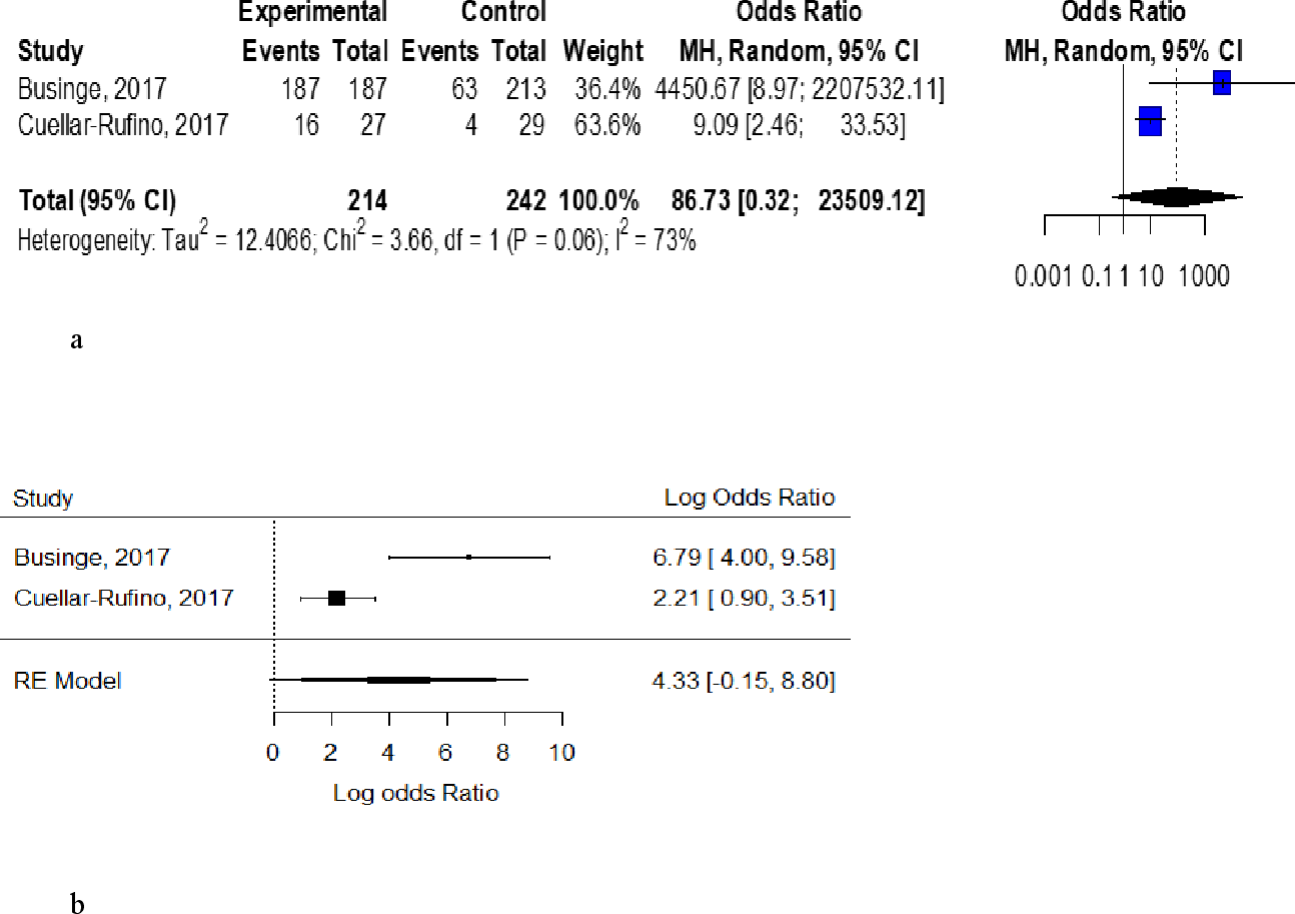
(A) Forest plot showing the odds of pre-eclampsia among women with urinary iodine concentration (UIC) <150 µg/L. (B) Forest plot showing the log odds of pre-eclampsia among women with UIC >150 µg/L (p=0.068, Tau^2^=9.8, I^2^=88.94%).

Two case–control studies had data with proportions of pre-eclamptic and normotensive participants with UIC above or below <150 µg/L.^22 23^ The odds of pre-eclampsia among women with UIC <150 µg/L were above unity for individual studies, but the pooled OR of 86.73 (0.32 - 23 509.12) was not significant with substantial heterogeneity across studies (I^2^=73%) (figure 3A, B).

**Figure 4:**
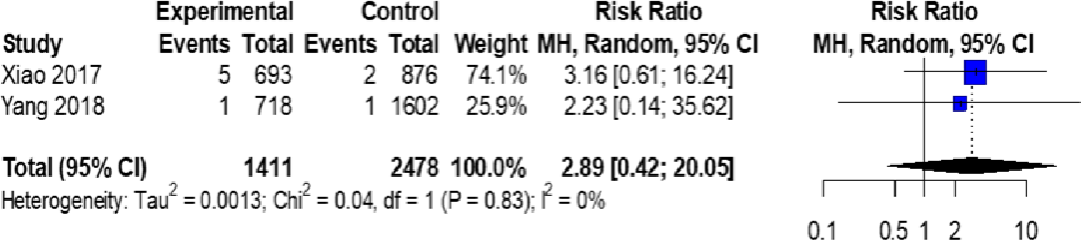
Forest plot showing the risk of pre-eclampsia among women with urinary iodine concentration <150 µg/L (designated as experimental group). Pooled risk ratio=2.89 (0.42 to 20.05), p=0.09.

The incidence of pre-eclampsia in the two cohort studies was 2/2320 and 7/1576, respectively.^26 27^ There was no difference in the incidence of pre-eclampsia among participants with or without low UIC (<150 µg/L) as shown in figure 4.

### Publication bias

**Figure 5:**
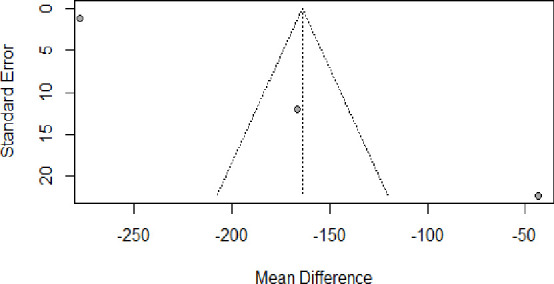
Funnel plot for the studies selected for the analysis of the mean difference in urinary iodine concentration of pre-eclamptic women and normotensive counterparts.

Visual inspection of funnel plot symmetry suggested potential publication bias for the studies included in the meta-analysis of UIC difference of pre-eclamptic and normotensive counterparts as well as the odds of pre-eclampsia among women with UIC <150 µg/L (figure 5).

**Figure 6:**
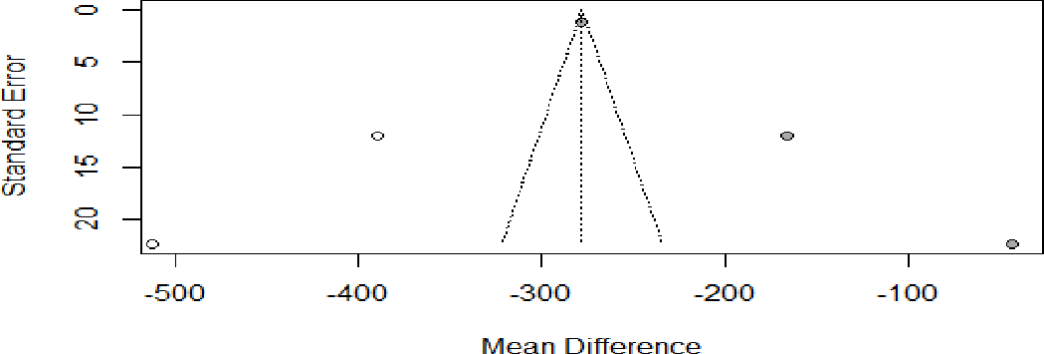
Funnel plots for publication bias in the studies selected for the analysis of the mean difference urinary iodine concentration of pre-eclamptic women and normotensive counterparts. The two imputed studies are represented by empty circles.

After adjustment of the effect size for potential publication bias using the trim-and-fill correction, two potentially missing studies (figure 6) were imputed in funnel plot (mean UIC differences of −389.60 (−413.02; −366.17) and −512.50 (−556.23; −468.78), respectively. With potential inclusion of the missing studies, the pooled mean UIC was estimated to be −278.0000 (−438.3025; −117.6975), which is significantly different from the pooled estimate of the three included studies (p<0.001).

**Figure 7:**
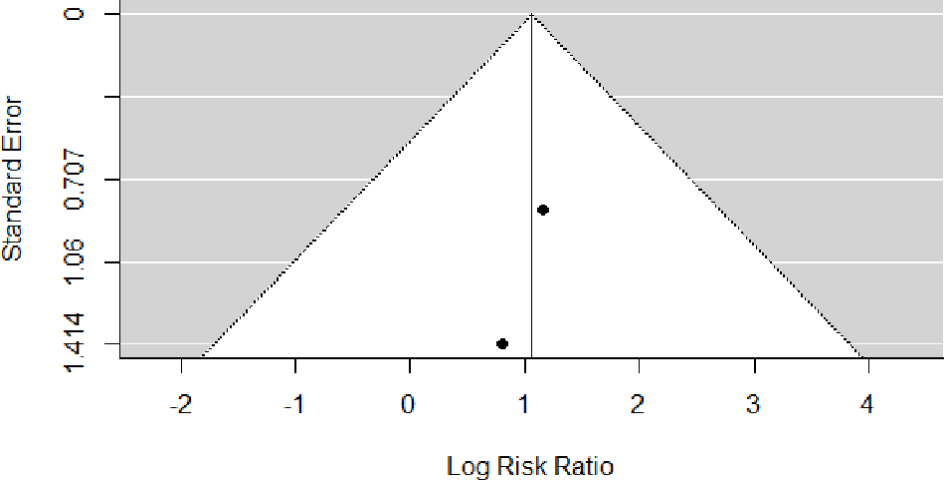
Funnel plot for the studies selected for the analysis of the risk of pre-eclampsia among women with urinary iodine concentration <150 µg/L.

The funnel plot for the cohort studies included in the assessment of the incidence of pre-eclampsia among women with UIC <150 µg/L was not suggestive of potential publication bias (figure 7).

## Discussion

The current review has shown that pre-eclamptic women have significantly lower mean UIC than their normotensive counterparts. This trend was observed in all the three included studies despite being from three different continents: Africa, Europe and South America.^23–25^ This association between low UIC and pre-eclampsia may reflect inadequate iodine intake predating pregnancy persisting until the third trimester that may increase the risk of pre-eclampsia among susceptible women. A recent Norwegian Study reported that among women with mild to moderate deficiency, long-term preconception iodine supplementation was associated with reduced incidence of pre-eclampsia.^7^

Although there was a trend towards a positive association between low UIC (<150 µg/L) in the third trimester and pre-eclampsia for the included case–control and cohort studies, the pooled OR and risk ratio showed a non-significant association. The small number of eligible studies that also had substantially high heterogeneity may partially account for this result. Hence, the available data are insufficient to provide a definitive answer on the risk of pre-eclampsia associated with low UIC in the third trimester.

Iodine deficiency is thought to predispose to incident pre-eclampsia through two mechanisms. The first one is the reduction of the antioxidant capacity of the placenta, which is one of the organs where the sodium iodine symporter maintains a high concentration of iodine, which, among other roles, is thought to reduce oxidative stress and lipid peroxide formation, which are elevated in patients with pre-eclampsia.^28 29^ The second mechanism is persistent iodine deficiency predisposing to elevated TSH. TSH operating via its endothelial receptors has been shown to diminish endothelial nitric oxide and prostacyclin production as well as upregulate endothelin production, which lead to endothelial dysfunction and systemic vasoconstriction.^30–32^ Since baseline prepregnancy as well as serial pregnancy UIC analyses were not carried out in the two cohort studies, it remains uncertain whether the iodine nutritional status at enrolment truly reflected the iodine nutritional status before pregnancy and for the remaining duration of the pregnancy following enrolment. Iodine nutritional status is likely to change with dietary habits and the progressive physiological changes of pregnancy. This could lead to misclassification of study participants and dilute the association between iodine deficiency and pre-eclampsia.^33^ The estimation of maternal intrathyroid iodine concentration, even though more technical, has been proposed as a more objective measure of prepregnancy iodine nutrition status than spot UIC.^34^ Concurrent measurement of spot UIC and serum thyroglobulin may help identify individuals with long-term exposure to iodine deficiency in studies where it is not possible to measure serial UIC and intrathyroid iodine concentration.^35 36^

### Limitations

This review was limited by the small number of eligible studies with small sample sizes and substantial degree of heterogeneity. The varied research designs of the eligible studies precluded the pooling of all the test results.

## Conclusion

Although the UIC of women who present with pre-eclampsia seems to be lower than that of women who remain normotensive until delivery, the available data are insufficient to reliably draw a conclusion on the association of iodine deficiency with the risk of pre-eclampsia. More well-designed and adequately powered studies that also include the estimation of prepregnancy iodine nutrition status are needed to address this question.

## Supplementary Material

Reviewer comments

Author's manuscript

## References

[1] Abalovich M, Gutierrez S, Alcaraz G, et al Overt and subclinical hypothyroidism complicating pregnancy. Thyroid 2002;12:63–8. 10.1089/105072502753451986 11838732

[2] Khanam M, Ilias M Study of thyroid hormonal status in preeclamptic patients. Med Today 2013;25:63–6. 10.3329/medtoday.v25i2.17922

[3] Wilson KL, Casey BM, McIntire DD, et al Subclinical thyroid disease and the incidence of hypertension in pregnancy. Obstet Gynecol 2012;119:315–20. 10.1097/AOG.0b013e318240de6a 22270283

[4] Lazarus JH Screening for thyroid dysfunction in pregnancy: is it worthwhile? J Thyroid Res 2011;2011:397012. 10.4061/2011/397012 21765989 PMC3134289

[5] Jameson LJ, Mandel SJ, Weetman AP Ch 405- disorders of the thyroid gland : Kasper DL, Hauser SL, Jameson JL, et al, Principles of internal medicine 19th ed. New York: McGraw Hill Education, 2015: 2283–308.

[6] Pharoah P, Buttfield IH, Hetzel BS Neurological damage to the fetus resulting from severe iodine deficiency during pregnancy. Int J Epidemiol 2012;41:589–92. 10.1093/ije/dys070 22586135

[7] Abel MH, Caspersen IH, Sengpiel V, et al Insufficient maternal iodine intake is associated with subfecundity, reduced foetal growth, and adverse pregnancy outcomes in the Norwegian mother, father and child cohort study. BMC Med 2020;18:211. 10.1186/s12916-020-01676-w 32778101 PMC7418397

[8] Andersson M, Karumbunathan V, Zimmermann MB Global iodine status in 2011 and trends over the past decade. J Nutr 2012;142:744–50. 10.3945/jn.111.149393 22378324

[9] Zimmermann MB Iodine deficiency in industrialized countries. Clin Endocrinol 2011;75:287–8. 10.1111/j.1365-2265.2011.04168.x 21729125

[10] De Groef B, Decallonne BR, Van der Geyten S, et al Perchlorate versus other environmental sodium/iodide symporter inhibitors: potential thyroid-related health effects. Eur J Endocrinol 2006;155:17–25. 10.1530/eje.1.02190 16793945

[11] Steinmaus C, Miller MD, Howd R Impact of smoking and thiocyanate on perchlorate and thyroid hormone associations in the 2001-2002 National health and nutrition examination survey. Environ Health Perspect 2007;115:1333–8. 10.1289/ehp.10300 17805424 PMC1964908

[12] Vidal ZEO, Rufino SC, Tlaxcalteco EH, et al Oxidative stress increased in pregnant women with iodine deficiency. Biol Trace Elem Res 2014;157:211–7. 10.1007/s12011-014-9898-6 24464603

[13] Moher D, Shamseer L, Clarke M, et al Preferred reporting items for systematic review and meta-analysis protocols (PRISMA-P) 2015 statement. Syst Rev 2015;4:1. 10.1186/2046-4053-4-1 25554246 PMC4320440

[14] WHO, UN Children’s Fund, International Council for the Control of Iodine Deficiency Disorders Assessment of iodine deficiency disorders and monitoring their elimination. A guide for programme managers. 3rd edn Geneva: World Health Organization, 2007.

[15] WHO (World Health Organization) Proceedings of the WHO technical consultation on control of iodine deficiency in pregnant women and young children. Geneva: WHO, 2005.

[16] Tranquilli AL, Dekker G, Magee L, et al The classification, diagnosis and management of the hypertensive disorders of pregnancy: a revised statement from the ISSHP. Pregnancy Hypertens 2014;4:97–104. 10.1016/j.preghy.2014.02.001 26104417

[17] Businge CB, Madini N, Longo-Mbenza B, et al Insufficient iodine nutrition status and the risk of pre-eclampsia: a protocol for systematic review and meta-analysis. BMJ Open 2019;9:e025573. 10.1136/bmjopen-2018-025573 PMC6538048 31129578

[18] Wells GA, Shea B, O’Connell D The Newcastle-Ottawa scale (NOS) for assessing the quality of nonrandomised studies in meta-analyses, 2018 Available: http://www.evidencebasedpublichealth.de/download/Newcastle_Ottawa

[19] McHugh ML Interrater reliability: the kappa statistic. Biochem Med 2012;22:276–82. 10.11613/BM.2012.031 PMC3900052 23092060

[20] Riley RD, Higgins JPT, Deeks JJ Interpretation of random effects meta-analyses. BMJ 2011;342:d549. 10.1136/bmj.d549 21310794

[21] Higgins JPT, Thompson SG, Deeks JJ, et al Measuring inconsistency in meta-analyses. BMJ 2003;327:557–60. 10.1136/bmj.327.7414.557 12958120 PMC192859

[22] Egger M, Davey Smith G, Schneider M, et al Bias in meta-analysis detected by a simple, graphical test. BMJ 1997;315:629–34. 10.1136/bmj.315.7109.629 9310563 PMC2127453

[23] Gulaboglu M, Borekci B, Delibas I Urine iodine levels in preeclamptic and normal pregnant women. Biol Trace Elem Res 2010;136:249–57. 10.1007/s12011-009-8539-y 19865803

[24] Cuellar-Rufino S, Navarro-Meza M, García-Solís P, et al Iodine levels are associated with oxidative stress and antioxidant status in pregnant women with hypertensive disease. Nutr Hosp 2017;34:661–6. 10.20960/nh.460 28627204

[25] Businge BC, Longo-Mbenza B, Adeniyi OV Iodine deficiency in pregnancy as a predictor of Sub-clinical hypothyroidism, preeclampsia and future cardiovascular disease. J Clin Nutri 2017;9:118–23.

[26] Yang J, Liu Y, Liu H, et al Associations of maternal iodine status and thyroid function with adverse pregnancy outcomes in Henan Province of China. J Trace Elem Med Biol 2018;47:104–10. 10.1016/j.jtemb.2018.01.013 29544795

[27] Xiao Y, Sun H, Li C, et al Effect of iodine nutrition on pregnancy outcomes in an Iodine-Sufficient area in China. Biol Trace Elem Res 2018;182:231–7. 10.1007/s12011-017-1101-4 28770411

[28] Burns R, O'Herlihy C, Smyth PPA The placenta as a compensatory iodine storage organ. Thyroid 2011;21:541–6. 10.1089/thy.2010.0203 21417918

[29] Brown SHJ, Eather SR, Freeman DJ, et al A lipidomic analysis of placenta in preeclampsia: evidence for lipid storage. PLoS One 2016;11:e0163972. 10.1371/journal.pone.0163972 27685997 PMC5042456

[30] Tian L, Zhang L, Liu J, et al Effects of TSH on the function of human umbilical vein endothelial cells. J Mol Endocrinol 2014;52:215–22. 10.1530/JME-13-0119 24444496

[31] Dardano A, Ghiadoni L, Plantinga Y, et al Recombinant human thyrotropin reduces endothelium-dependent vasodilation in patients monitored for differentiated thyroid carcinoma. J Clin Endocrinol Metab 2006;91:4175–8. 10.1210/jc.2006-0440 16868055

[32] Lioudaki E, Mavroeidi NG, Mikhailidis DP, et al Subclinical hypothyroidism and vascular risk: an update. Hormones 2013;12:495–506. 10.14310/horm.2002.1437 24457397

[33] Haine D, Dohoo I, Dufour S Selection and misclassification biases in longitudinal front. Vet Sci 2018;5:99. 10.3389/fvets.2018.00099 PMC5985700 29892604

[34] Dineva M, Fishpool H, Rayman MP, et al Systematic review and meta-analysis of the effects of iodine supplementation on thyroid function and child neurodevelopment in mildly-to-moderately iodine-deficient pregnant women. Am J Clin Nutr 2020;112:389–412. 10.1093/ajcn/nqaa071 32320029

[35] Du Y, Gao YH, Feng ZY, et al Serum Thyroglobulin-A sensitive biomarker of iodine nutrition status and affected by thyroid abnormalities and disease in adult populations. Biomed Environ Sci 2017;30:508–16. 10.3967/bes2017.067 28756810

[36] Bath SC, Pop VJM, Furmidge-Owen VL, et al Thyroglobulin as a functional biomarker of iodine status in a cohort study of pregnant women in the United Kingdom. Thyroid 2017;27:426–33. 10.1089/thy.2016.0322 27762729 PMC5337401

