# Supplementary material for: Insufficient iodine nutrition status and the risk of pre-eclampsia: a systemic review and meta-analysis
Source: BMJ Open. 2021 Feb 10;11(2):e043505. doi: 10.1136/bmjopen-2020-043505 (PMC7878161; doi:10.1136/bmjopen-2020-043505)
Supplement: Author's manuscript [file bmjopen-2020-043505.draft_revisions.pdf]

BMJ Open

BMJ Open is committed to open peer review. As part of this commitment we make the peer review history of every article we publish publicly available.

When an article is published we post the peer reviewers' comments and the authors' responses online. We also post the versions of the paper that were used during peer review. These are the versions that the peer review comments apply to.

The versions of the paper that follow are the versions that were submitted during the peer review process. They are not the versions of record or the final published versions. They should not be cited or distributed as the published version of this manuscript.

BMJ Open is an open access journal and the full, final, typeset and author-corrected version of record of the manuscript is available on our site with no access controls, subscription charges or pay-per-view fees (http://bmjopen.bmj.com).

If you have any questions on BMJ Open's open peer review process please email info.bmjopen@bmj.com

BMJ Open

Insufficient iodine nutrition status and the risk of preeclampsia: a systemic review and meta-analysis

Journal:	*BMJ Open*
Manuscript ID	bmjopen-2020-043505
Article Type:	Original research
Date Submitted by the Author:	12-Aug-2020

Complete List of Authors:	Businge, Charles; University of Cape Town, Department of Medicine; Walter Sisulu University Faculty of Health Sciences, Department of Obstetrics and Gynaecology<br>Usenbo, Anthony; Nelson Mandela Academic Hospital, Department of Anesthesiology<br>Longo-Mbenza, Benjamin; University of Kinshasa, Faculty of Medicine,; Lomo University of Research, Lomo Research Laboratory<br>Kengne , AP; South African Medical Research Council,

Keywords:	Maternal medicine < OBSTETRICS, EPIDEMIOLOGY, NUTRITION & DIETETICS

SCHOLARONE™
Manuscripts

I, the Submitting Author has the right to grant and does grant on behalf of all authors of the Work (as defined in the below author licence), an exclusive licence and/or a non-exclusive licence for contributions from authors who are: i) UK Crown employees; ii) where BMJ has agreed a CC-BY licence shall apply, and/or iii) in accordance with the terms applicable for US Federal Government officers or employees acting as part of their official duties; on a worldwide, perpetual, irrevocable, royalty-free basis to BMJ Publishing Group Ltd ("BMJ") its licensees and where the relevant Journal is co-owned by BMJ to the co-owners of the Journal, to publish the Work in this journal and any other BMJ products and to exploit all rights, as set out in our licence.

The Submitting Author accepts and understands that any supply made under these terms is made by BMJ to the Submitting Author unless you are acting as an employee on behalf of your employer or a postgraduate student of an affiliated institution which is paying any applicable article publishing charge ("APC") for Open Access articles. Where the Submitting Author wishes to make the Work available on an Open Access basis (and intends to pay the relevant APC), the terms of reuse of such Open Access shall be governed by a Creative Commons licence – details of these licences and which Creative Commons licence will apply to this Work are set out in our licence referred to above.

Other than as permitted in any relevant BMJ Author's Self Archiving Policies, I confirm this Work has not been accepted for publication elsewhere, is not being considered for publication elsewhere and does not duplicate material already published. I confirm all authors consent to publication of this Work and authorise the granting of this licence.

For peer review only

Insufficient iodine nutrition status and the risk of preeclampsia: a systemic review and meta-analysis

*Charles Bitamazire Businge[1, 2], Anthony Usenbo[3], Benjamin Longo-Mbenza[4], Andre Pascal Kengne[2, 5]

[1]Department of Obstetrics and Gynaecology, Faculty of Health Sciences, Walter Sisulu University, Private Bag x1 WSU, 5117, Mthatha, South Africa

[2]Department of Medicine, Faculty of Health Sciences, University of Cape Town, Cape Town, South Africa

Email: bsnbit001@myuct.ac.za /cbusingae@gmail.com /cbusinge@wsu.ac.za

[3]Nelson Mandela Academic Hospital, Mthatha, Eastern Cape, South Africa.

Email: tonyusenbo@gmail.com

[4]Faculty of Medicine, University of Kinshasa, Democratic Republic of Congo and LOMO University of Research, Kinshasa, Democratic Republic of Congo

Email: longombenza@gmail.com / longombenza@yahoo.fr

[5]Non-Communicable Disease Research Unit, South African Medical Research Council, Cape Town, South Africa
Email: Andre.Kengne@mrc.ac.za

*Corresponding author

Word count - 3892

For peer review only

Abstract

Background: Although sub-clinical hypothyroidism (SCH) in pregnancy is one of the established risk factors for preeclampsia, the link between iodine deficiency, the main cause of hypothyroidism, and preeclampsia remains uncertain. We conducted a systematic review to determine the iodine nutrition status of pregnant women with and without preeclampsia and the risk of preeclampsia due to iodine deficiency.

Methods: MEDLINE, EMBASE, Google Scholar, SCOPUS and Africa Wide Information were searched up to June 30th 2020. Random-effects models meta-analysis was used to pool mean difference UIC between preeclamptic and normotensive controls; and pool odds ratios and incidence rates of preeclampsia among women with UIC <150 μg/L.

Results: Five eligible studies were included in the meta-analysis. There was a significant difference in the pooled mean UIC of 254 preeclamptic women and 210 normotensive controls enrolled in eligible 3 cross-sectional studies (mean UIC 164.4 μg/L [95% CI 45.1 – 283.6, $p < 0.01$, $I^2 > 50$]). The overall proportions of preeclampsia among women with UIC <150 μg/L and UIC >150 μg/L in two cross-sectional studies were 203/214 and 67/247 respectively with a pooled odds ratio of 0.01 (95% CI 0.00 – 4.23, $p = 0.14$, $I^2 > 50$) for preeclampsia among women with UIC >150 μg/L. The overall incidence of preeclampsia among women with UIC <150 μg/L and UIC >150 μg/L in two cohort studies were 6/1411 and 3/2478 respectively with a pooled risk ratio of 2.85 (95% CI 0.42 – 20.05, $p = 0.09$, $I^2 < 25$)

Conclusion: Although preeclamptic women seem to have lower UIC than normotensive pregnant women, the available data is insufficient to provide a conclusive answer on association of iodine deficiency with preeclampsia risk.

Key words

Iodine deficiency, pregnancy, preeclampsia

Strengths and limitations of this study

- The current study is among the first systematic reviews that have ascertained the relationship between insufficient iodine nutrition status and pre-eclampsia.
- This review was however been limited by the small number of eligible studies coupled with small sample sizes.

For peer review only

For peer review only - http://bmjopen.bmj.com/site/about/guidelines.xhtml

- The varied study designs coupled with a considerable degree of heterogeneity precluded the pooling of all the results.

This review is registered with PROSPERO (registration no. CRD42018099427).

For peer review only

For peer review only - http://bmjopen.bmj.com/site/about/guidelines.xhtml

INTRODUCTION

Sub-clinical hypothyroidism (SCH) is a risk factor for preeclampsia, which is a prominent cause of maternal and perinatal morbidity and mortality. [1-3] Iodine deficiency, which is exacerbated by pregnancy related physiological changes, is a leading cause of hypothyroidism.[4-5] As a consequence, insufficient iodine intake in pregnancy could be a risk factor of preeclampsia, particularly in endemic iodine deficiency settings.

Over two billion people live in areas with iodine insufficiency.[6] In addition, iodine deficiency is on the rise in areas originally thought to be iodine sufficient, despite concerted worldwide efforts to promote iodine fortification. This has been attributed in part to high concentration of perchlorate and thiocyanate in water sources and the diet, which impairs the uptake of iodine by the thyroid gland, particularly among individuals with TSH-related thyroid stimulation secondary to iodine deficiency.[7-10]

In this systematic review and meta-analysis, we sought to establish if there a difference in the urinary iodine concentration (UIC) of pregnant women with and without pre-eclampsia; and whether pregnant women with insufficient iodine nutrition status are at increased risk of pre-eclampsia.

The study is reported according to the Preferred Reporting Items for Systematic reviews and Meta-Analysis (PRISMA) Guidelines, [11] and was based on a protocol that was registered with the International Prospective Register of Systematic Reviews, PROSPERO (registration no. CRD42018099427).

Methods

Eligibility criteria

Inclusion criteria

The selection of studies for inclusion was guided by the Population, Intervention/exposure, Comparison and Outcome (PICO) protocol. The target population were pregnant women and the exposure was insufficient iodine nutrition status before pregnancy for cohort studies, and insufficient iodine nutrition status during pregnancy for case-control studies. The iodine nutrition status was defined according to the World Health Organization / International Council for Control of Iodine Deficiency Disorders (ICCIDD) classification of iodine intake using median urinary iodine concentration (UIC).[12] [13] For pregnant women, a urine iodine concentration (UIC) <150, 150-249, 250-499 and >500µg μgL^{-1} is considered an estimate of

For peer review only - http://bmjopen.bmj.com/site/about/guidelines.xhtml

insufficient, adequate, more than adequate and excessive iodine nutritional status, respectively.[13] The comparators were study participants with sufficient iodine nutrition status (UIC >150 µg/L) during pregnancy for case-control studies and UIC >100 µg/L before the onset of pregnancy for cohort studies.[12 13] The outcomes were the prevalence and incidence rates of preeclampsia among women with and without adequate iodine nutrition status from which the odds ratios for case control and risk ratios for cohort studies were determined.

Preeclampsia was defined as new onset hypertension after 20 weeks of amenorrhoea characterised by elevated systolic blood pressure (SBP ≥140 mmHg) and/or diastolic blood pressure (DBP ≥90 mmHg), based on two measurements four hours apart; or SBP>160 mmHg and/or DBP>110 mmHg from a single measurement. Elevated BP had to be accompanied by at least one of the following: proteinuria in 24 h-urine ≥ 300 mg or protein/creatinine ratio ≥ 0.3 or urine protein measured by dipstick ≥ 1+; thrombocytopenia (platelets count less than 150,000/µL), kidney insufficiency (serum creatinine levels above 97 µmol/L), decreased liver function (AST and ALT twice higher than the upper limit of the reference interval), compromised lung function or pulmonary oedema, visual or other symptoms and signs of impaired cerebral function.[14] There may be considerable heterogeneity if preeclampsia has been variably defined in different studies that are eligible for inclusion in the current systematic review.

Exclusion criteria

Studies were excluded if they lacked means, medians, odds ratios, incidence and prevalence rates data to compute them even after repeated unsuccessful attempts to contact the authors via email for relevant information. Letters to editors, reviews, commentaries, editorials and any publication without primary data were also excluded.

Patient and public involvement

There was no involvement of the public or patients.

Search Strategy and Selection Criteria

We searched PubMed, Scopus, Web of Science, Academic Search Premier, Africa Wide Information, CINAHL, Cochrane Library, Google Scholar, and Health Source: Nursing/Academic Edition databases for all published studies on iodine deficiency and preeclampsia up to 30th June 2020. This search was conducted using a predefined comprehensive and sensitive search strategy (Table 1) combining relevant terms and synonyms

For peer review only - http://bmjopen.bmj.com/site/about/guidelines.xhtml

which are variably used to denote abnormally high blood pressure in pregnancy and insufficient iodine intake or iodine deficiency as detailed in the published protocol for this review.[15]

Study Selection and Data Extraction

Two authors (CBB and AU) independently screened the titles and abstracts of identified studies. Citations and abstracts were initially screened and duplicate citations excluded. Titles and abstracts were then screened following inclusion criteria described in the protocol[15], after which the full-texts of potentially eligible articles were obtained. These full texts were screened using a standardised and pretested form to include eligible studies. Disagreements were resolved by consensus. For each study, one reviewer (CBB) extracted the data and a second reviewer (AU) checked the accuracy. For the 5 studies included here, there were no disagreements between the two reviewers. Figure 1 shows the flowchart for the selection process. The following data were extracted from the eligible studies: studies characteristics (authors, years, design, and study regions), study population (age, and sample size), iodine nutrition status of the various study groups, and the methods of outcomes measurement.

Quality Assessment

Two reviewers (CBB and AU) independently scored the risk of bias and the quality of included studies (Table 2a and Table 2b) using the Newcastle-Ottawa scale. [16] Inter-rater agreement on screening, data abstraction and methodological quality (selection, comparability of groups and ascertainment of exposure/outcome) was assessed using Cohen's κ coefficient. [17] The Kappa value for interrater agreement for quality assessment was 0.694 ($p < 0.001$). Discrepancies were resolved by consensus.

Data synthesis, analysis and assessment of heterogeneity

The analysis was performed with the Review Manager (RevMan) Software, version 5.4 (The Nordic Cochrane Centre, The Cochrane Collaboration) and the 'meta' and 'metafor' packages of the statistical software R (version 4.0.2, The R Foundation for statistical computing, Vienna, Austria). For the outcomes of interest (means, prevalence and incidence rates), random effects model meta-analyses were used to pool estimates across studies with similar design. [18] The degree of heterogeneity across studies was assessed using the Cochrane Q statistic and inconsistency index (I^2) statistic and values ranked as indicating low: I^2<25%; moderate: 25%–

For peer review only - http://bmjopen.bmj.com/site/about/guidelines.xhtml

50%; high heterogeneity: I^2>50%).[19] The Egger funnel plot was used to check for publication bias.[20]

Results

The review process

The process for selecting the relevant studies is summarised in Figure 1. In total, 2380 records were identified via database searches. After removing duplicates, we scanned the titles and abstracts of 522 articles, of which 11 full texts were further reviewed. Of these, 5 articles met criteria for inclusion in the current systematic review.

Characteristics of included studies

All the 5 included studies were categorised as having a low risk of bias. Their characteristics are summarised in Table 3. Three were institutional-based case-control studies one from each of the following countries: Turkey, Mexico and the Democratic Republic of Congo while 2 were prospective cohort studies were from two different provinces (Henan and Liaoning) in China.[21 - 25]

Meta-analysis

Mean difference in urinary iodine concentration of preeclamptic and normotensive women

Three studies reported the mean UIC of preeclamptic and normotensive pregnant women.[21 – 23] Overall there was a significant and positive mean difference in UIC and standardised mean UIC of normotensive pregnant women and preeclamptic women, with substantial heterogeneity across studies (Fig 2).

The risk of preeclampsia among women with UIC < 150 µg/L

Two cross-sectional studies had data with proportions of preeclamptic and normotensive participants with UIC above or below <150 µg/L.[22 23] The odds of preeclampsia among women with UIC<150 µg/L was above unity for individual studies but the pooled odds ratio of 86.73 (0.32, 23509.12) was not significant with substantial heterogeneity across studies (I^2 = 73%) (Fig 3a and 3b).

The incidence of preeclampsia in the two cohort studies was 2/2320 and 7/1576 respectively.[24 25] There was no difference in the incidence of preeclampsia among participants with or without low UIC (UIC <150µg/L) as shown in Figure 4.

For peer review only - http://bmjopen.bmj.com/site/about/guidelines.xhtml

Publication Bias

Visual inspection of funnel plot symmetry suggested potential publication bias for the studies included in the meta-analysis of UIC difference preeclamptic and normotensive counterparts as well as the odds of preeclampsia among women with <150 µg/L.

After adjustment of the effect size for potential publication bias using the trim-and-fill correction, 2 potentially missing studies (Figure 6) were imputed in funnel plot (Mean UIC differences of -389.60 [-413.02; -366.17] and -512.50 [-556.23; -468.78] respectively. With potential inclusion of the missing studies the pooled mean UIC was estimated to be -278.0000 [-438.3025; -117.6975] which is significantly different from the pooled estimate of the 3 included studies (p <0.001).

Discussion

The current review has shown that preeclamptic women have significantly lower mean UIC than their normotensive counterparts. This trend was observed in all the 3 included studies despite being from 3 different continents: Africa, Europe and South America.[21-23] This association between low UIC and preeclampsia may reflect inadequate iodine intake persisting till the third trimester that may increase the risk of preeclampsia among susceptible women.

Although there was a trend towards a positive association between low UIC (UIC<150µg/L) in the third trimester and preeclampsia for the included case-control and cohort studies, the pooled odds and risk ratios showed a non-significant association. The small number of eligible studies which also had substantially high heterogeneity may partially account for this result. Hence the available data is insufficient to provide a definitive answer on the risk of preeclampsia associated with low UIC in the third trimester.

Iodine deficiency is thought to predispose to incident preeclampsia through two mechanisms. The first one is the reduction of the anti-oxidant capacity of the placenta, which is one of the organs where the sodium iodine symporter maintains a high concentration of iodine which among other roles, is thought to reduce oxidative stress and lipid peroxide formation which are elevated in patients with preeclampsia.[26–28] The second mechanism is persistent iodine deficiency predisposing to elevated TSH. TSH operating via its endothelial receptors has been shown to diminish endothelial Nitric Oxide and prostacyclin production as well as upregulate

For peer review only - http://bmjopen.bmj.com/site/about/guidelines.xhtml

endothelin production, which lead to endothelial dysfunction and systemic vasoconstriction.[29-31] Since serial UIC analyses were not carried out in the two cohort studies, it remains uncertain whether the iodine nutritional status at enrolment truly reflected the iodine nutritional status for the remaining duration of the pregnancy. Iodine nutritional status is likely to change with dietary habits and the progressive physiological changes of pregnancy. This could lead to misclassification of study participants, and dilute the association between iodine deficiency and preeclampsia.[32] Concurrent measurement of spot UIC and serum thyroglobulin may help identifying individuals with long term exposure to iodine deficiency in studies where it is not possible to measure serial UIC.[33 34]

Limitations

This review was limited by the small number of eligible studies with small sample sizes and substantial degree of heterogeneity. The varied research designs of the eligible studies precluded the pooling of all the test results.

Conclusion

Although the UIC of women who present with preeclampsia seems to be lower than that of women who remain normotensive till delivery, the available data is insufficient to reliably draw a conclusion on the association of iodine deficiency with the risk of preeclampsia. More well-designed and adequately powered studies are needed to address this question.

Funding

This study is part of a research project supported by the Discovery Foundation Rural Fellowship Grant number 038372. APK is employed by the South African Medical Research Council.

Competing interests

There are no competing interests.

Patient consent for publication

This not applicable since the current study is a systematic review and meta-analysis of already published data.

Figure captions

Figure 1: Study selection flowchart

Figure 2: Forest plot showing the mean difference in UIC of normotensive and preeclamptic mothers

For peer review only - http://bmjopen.bmj.com/site/about/guidelines.xhtml

Figure 3a: Forest plot showing the odds of preeclampsia among women with UIC < 150 µg/L

Figure 3b: Forest plot showing the Log odds of preeclampsia among women with UIC > 150 µg/L. p = 0.068, $Tau^2 = 9.8$, $I^2 = 88.94\%$.

Figure 4: Forest plot showing the risk of preeclampsia among women with <150 µg/L (designated as experimental group). Pooled RR 2.89 (0.42 – 20.05), p = 0.09.

Figure 5: Funnel plot for the studies selected for the analysis of the mean difference UIC of preeclamptic women and normotensive counterparts

Figure 6: Funnel plots for publication bias in the studies selected for the analysis of the mean difference UIC of preeclamptic women and normotensive counterparts. The 2 imputed studies are represented by empty circles.

Figure 7: Funnel plot for the studies selected for the analysis of the risk of preeclampsia among women with <150 µg/L.

References

1. Abalovich M, Gutierrez S, Alcaraz G, *et al*. Overt and subclinical hypothyroidism complicating pregnancy. *Thyroid* 2002;12(1): 63-68.

2. Khanam M, Ilias M. Study of Thyroid Hormonal Status in Preeclamptic Patients. *Medicine Today* 2013;25(2): 63 - 66.

3. Wilson KL, Casey BM, McIntire DD, *et al*. Subclinical thyroid disease and the incidence of hypertension in pregnancy. *Obstet Gynecol*, 2012;119(2 Pt 1):315-20.

4. Lazarus JH. Screening for Thyroid function in Pregnancy: Is It worthwhile? Journal of thyroid Research. [Online] 2011.Available: http://.dx.doi.org/10.4061/2011/397012.

5. Jameson LJ, Mandel SJ, Weetman AP. Ch 405- Disorders of the Thyroid gland. In: Kasper DL, Hauser SL, Jameson JL, Fauci AS, Longo DL, Loscalzo J, editors. Principles of Internal Medicine 19th Ed. New York: McGraw Hill Education, 2015; p 2283 - 2308.

6. Andersson M, Karumbunathan V, Zimmermann MB. Global iodine status in 2011 and trends over the past decade. *Journal of Nutrition* 2012;142(4):744-750.

For peer review only - http://bmjopen.bmj.com/site/about/guidelines.xhtml

7. Zimmermann MB. Iodine deficiency in industrialized countries. *Clinical Endocrinology* 2011;75: 287-288.

8. De Groef, B, Decallonne BR, Van der Geyten S, *et al*. Perchlorate versus other environmental sodium/iodide symporter inhibitors: potential thyroid related health effects. *Eur J Endocrinol* 2006;155(1):17-25.

9. Steinmaus C, Miller MD. Howd R. Impact of smoking and thiocyanate on perchlorate and thyroid hormone associations in the 2001-2002 national health and nutrition examination survey. *Environ Health Perspect* 2007;115(9):1333-8.

10. Vidal, ZEO, Rufino SC, Tlaxcalteco EH, *et al*. Oxidative Stress Increased in Pregnant Women with Iodine Deficiency. *Biol Trace Elem Res* 2014;157: 211.

11. Moher, D., Shamseer, L., Clarke, M., et al. Preferred reporting items for systematic review and meta-analysis protocols (PRISMA-P) 2015 statement. Syst Rev 2015;4:1.

12. WHO, UN Children's Fund, International Council for the Control of Iodine Deficiency Disorders. Assessment of iodine deficiency disorders and monitoring their elimination. A guide for programme managers, 3rd edn. Geneva: World Health Organization, 2007.

13. WHO (World Health Organization). Proceedings of the WHO Technical Consultation on control of iodine deficiency in pregnant women and young children. Geneva: WHO, 2005.

14. American College of Obstetricians and Gynecologists. Task Force on Hypertension in Pregnancy. Hypertension in Pregnancy. Report of the American College of Obstetricians and Gynecologists' Task Force on Hypertension in Pregnancy. Obstet Gynecol 2013; 122(5): 1122−31.

15. Businge CB, Madini N, Longo-Mbenza B, Kengne AP. Insufficient iodine nutrition status and the risk of pre-eclampsia: a protocol for systematic review and meta-analysis. BMJ Open. 2019 May;9(5):e025573. DOI: 10.1136/bmjopen-2018-025573.

16. Wells GA, Shea B, O'Connell D, Peterson J, Welch V, Losos M, et al. The Newcastle-Ottawa Scale (NOS) for assessing the quality of nonrandomised studies in meta-analyses [cited 2018 Oct 06]. Available from: http://www.evidencebasedpublichealth.de/download/Newcastle_Ottawa.

17. McHugh ML. Interrater reliability: the kappa statistic. Biochem Med (Zagreb), 2012;22(3): 276–282.

18. Riley RD, Higgins JPT, Deeks JJ. Interpretation of random effects meta-analyses. BMJ 2011;342:d549.

For peer review only - http://bmjopen.bmj.com/site/about/guidelines.xhtml

19. Higgins JP, Thompson SG, Deeks JJ, et al. Measuring inconsistency in meta-analyses. BMJ, 2003;327:557-60.

20. Egger M, Davey SG, Schneider M, Minder C. Bias in meta-analysis detected by a simple, graphical test. BMJ 1997;315:629.

21. Gulaboglu M, Borekci B, Delibas I. Urine iodine levels in preeclamptic and normal pregnant women. Biol Trace Elem Res 2010;136:249–257.

22. Cuellar-Rufino S, Navarro-Meza M, García-Solís P, Xochihua-Rosas I, Arroyo-Helguera O. Iodine levels are associated with oxidative stress and antioxidant status in pregnant women with hypertensive disease. Nutr Hosp. 2017 Jun 5;34(3):661-666.

23. Businge BC, Longo-Mbenza B, Adeniyi OV, Nzuzi Babeki V, Kitambala Kaboka A, Mvitu Muaka M, Nkanga Nganga SM, Tsobo CM, Vangu R, Diambu EM and l Ter Goon D. Iodine Deficiency in Pregnancy as a Predictor of Sub-clinical Hypothyroidism, Preeclampsia and Future Cardiovascular Disease. J Clin Nutri 2017; 9:118-123.

24. Yang J, Liua Y, Liub H, Zhenga H, Lia X, Zhua L, WangA Z. Associations of maternal iodine status and thyroid function with adverse pregnancy outcomes in Henan Province of China. J Trace Elements Med Biol 2018; 47:104–110.

25. Xiao Y, Sun H, Li C, Li Y, Peng S, Fan C, Teng W, Shan Z. Effect of Iodine Nutrition on Pregnancy Outcomes in an Iodine-Sufficient Area in China. Biol Trace Elem Res 2018; 182:231–237.

26. Burns R, O'Herlihy C, Smyth PP. The placenta as a compensatory iodine storage organ. Thyroid 2011;21(5):541-6.

27. Vidal ZEO, Rufino SC, Tlaxcalteco EH, et al. Oxidative Stress Increased in Pregnant Women with Iodine Deficiency. Biol Trace Elem Res 2014;157: 211.

28. Brown SHJ, Eather SR, Freeman DJ, Meyer BJ, Mitchell TW. A Lipidomic Analysis of Placenta in Preeclampsia: Evidence for Lipid Storage. PLoS ONE 2016; 11(9): e0163972. doi:10.1371/journal.pone.0163972.

29. Tian, L., Zhang, L., Liu, J. Guo, T., Gao, C. and Ni1, J. Effects of TSH on the function of human umbilical vein endothelial cells. Journal of Molecular Endocrinology 2014; 52: 215-222.

30. Dardano, A., Ghiadoni, L., Plantinga, Y., Caraccio, N., Bemi, A., Duranti, E., Taddei, S., Ferrannini, E., Salvetti, A. and Monzani, F. Recombinant Human Thyrotropin Reduces Endothelium-Dependent Vasodilation in Patients Monitored for Differentiated Thyroid Carcinoma. J Clin Endocrinol Metab 2006; 91: 4175-4178.

For peer review only - http://bmjopen.bmj.com/site/about/guidelines.xhtml

31. Lioudaki, E., Mavroeidi, N.G., Mikhailidis, D.P., Emmanouel S. Ganotakis, E.S. Subclinical hypothyroidism and vascular risk: An update. Hormones 2013; 12(4):495-506.

32. Haine D, Dohoo I, Dufour S. Selection and Misclassification Biases in Longitudinal Front. Vet. Sci. 2018; 5:99. doi: 10.3389/fvets.2018.00099.

33. Du Y, Gao YH, Feng ZY, Meng FG, Fan LJ, Sun DJ. Serum Thyroglobulin—A Sensitive Biomarker of Iodine Nutrition Status and Affected by Thyroid Abnormalities and Disease in Adult Populations. Biomed Environ Sci, 2017; 30(7): 508-516.

34. Bath SC, Pop VJM, Furmidge-Owen VL, Broeren MAC, Rayman MP.Thyroglobulin as a Functional Biomarker of Iodine Status in a Cohort Study of Pregnant Women in the United Kingdom. Thyroid 2017; 27(3):426-433.

Table 1 Search strategy for MEDLINE [15]

**Population: Pregnant women with Pre-eclampsia**		
#1	MeSH terms	Pregnant Women [Mesh] OR Pregnancy [Mesh] OR Pregnancy Trimesters [Mesh]
#2	Free text	Pregnancy OR Pregnant women OR expectant mothers
#3	#1 OR #2	
#4	MeSH terms	Pre-Eclampsia [Mesh] OR Eclampsia [Mesh] OR Hypertension [Mesh]
#5	Free text	Preeclampsia OR Pre-eclampsia OR Eclampsia OR Hypertension OR Hypertensive OR High blood pressure
#6	#4 OR #5	
**Exposure: Iodine deficiency**		
#7	MeSH terms	Iodine [Mesh]
#8	Free text	Iodine
#9	#7 OR #8	
#10	#3 AND #6 AND #9	

For peer review only - http://bmjopen.bmj.com/site/about/guidelines.xhtml

Table 2a: Risk of bias assessment (Reviewer CBB)

Study	Study type	Selection	Comparability	Outcome/exposure	≠AHRQ scale (good/fair/poor)
Gulaboglu, 2009	Case control	***	**	***	Good
Cuellar-Rufino, 2017	Case control	****	**	***	Good
Businge, 2017	Case control	****	**	**	Good
Yang, 2018	Cohort	****	**	***	Good
Xiao, 2017	Cohort	****	**	***	Good

≠AHQR = Agency for Healthcare Research and Quality

Table 2b: Risk of bias assessment (Reviewer AU)

Study	Study Type	Selection	Comparability	Outcome/ Exposure	≠AHRQ scale (good/fair/poor)
Gulaboglu 2009	Case-control	***	**	***	Good
Cuellar-Rufino, 2017	Case-control	***	**	**	Good
Businge 2017	Case-control	****	**	***	Good
Yang 2018	Cohort study	****	**	***	Good
Xiao 2017	Cohort study	****	**	***	Good

≠AHQR = Agency for Healthcare Research and Quality

For peer review only - http://bmjopen.bmj.com/site/about/guidelines.xhtml

413

414 Table 3: Characteristics of included studies

First author, year	Country	Study design	Study period	Cases(n)	Controls(n)	Comparator	Diagnostic criteria
**Gulaboglu, 2009**	Turkey	Case-control	Not stated	Severe preeclampsia (40)	Normotensives (18)	Mean IUC	Sandell–Kolthoff reaction
**Cuellar-Rufino, 2017**	Mexico	Case-control	Jan – April 2015	Preeclampsia (20)	Normotensives (37)	• Mean IUC<br>• IUC<150 µg/L	Fast colorimetric method
**Businge, 2017**	Congo Democratic Republic	Case-control	Jan 2007 to December 2008	Preeclampsia (68) and Severe preeclampsia/ Eclampsia (182)	Normotensives (150)	• Mean IUC<br>• IUC<150 µg/L	Sandell–Kolthoff reaction
**Yang, 2018**	Henan Province, China	cohort	July to September 2015	• Incident preeclampsia 01/718 for women with IUC <150 µg/L<br>• Incident gestational HT 17/718 for women with IUC <150 µg/L	• Incident preeclampsia 01/1602 for women with IUC >150 µg/L<br>• Incident gestational HT 25/1602 for women with IUC >150 µg/L	• IUC<150 µg/L	Fast colorimetric method
**Xiao, 2017**	Liaoning province, China	Cohort	2012 - 2014	• Incident preeclampsia 05/693 for women with IUC <150 µg/L<br>• Incident gestational HT 18/693 for women with IUC <150 µg/L	• Incident preeclampsia 02/876 for women with IUC >150 µg/L<br>• Incident gestational HT 25/876 for women with IUC >150 µg/L	• IUC<150 µg/L	Sandell–Kolthoff reaction

415

416

For peer review only - http://bmjopen.bmj.com/site/about/guidelines.xhtml

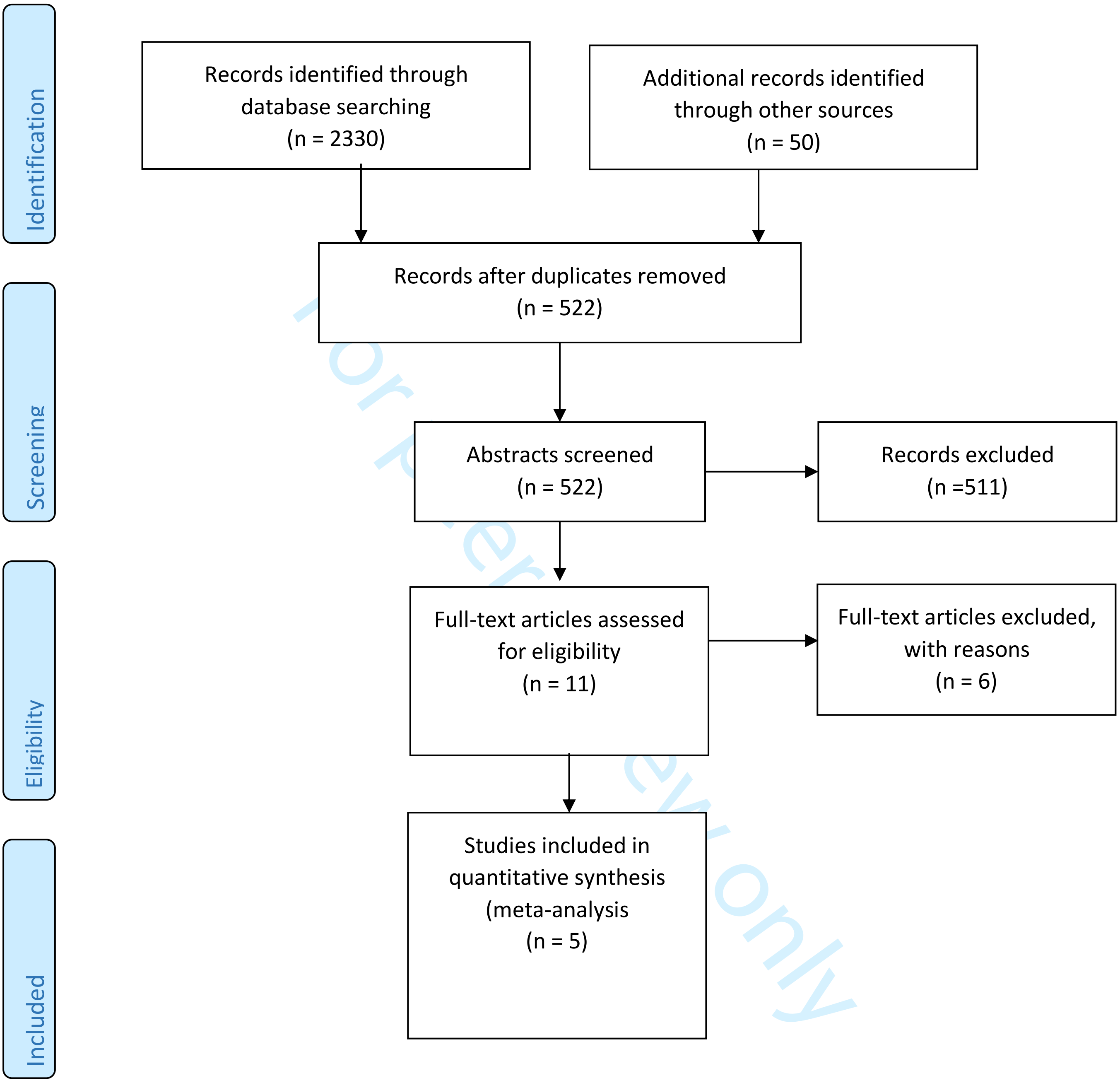

Figure 1: Study selection flowchart

Study or Subgroup	Normotensive controls Mean	SD	Total	Preeclamptics Mean	SD	Total	Weight	Mean Difference IV, Random, 95% CI
Cuellar-Rufino 2017	185.7	77.2	20	142.2	84.8	37	32.5%	43.50 [0.01, 86.99]
Gulaboglu 2009	208.9	64	40	42.5	27	18	33.5%	166.40 [142.97, 189.83]
Businge 2017	423.9	13	150	145.9	7.5	200	34.0%	278.00 [275.67, 280.33]
**Total (95% CI)**			**210**			**255**	**100.0%**	**164.36 [45.14, 283.58]**

Heterogeneity: Tau² = 10891.98; Chi² = 196.65, df = 2 (P < 0.00001); I² = 99%
Test for overall effect: Z = 2.70 (P = 0.007)

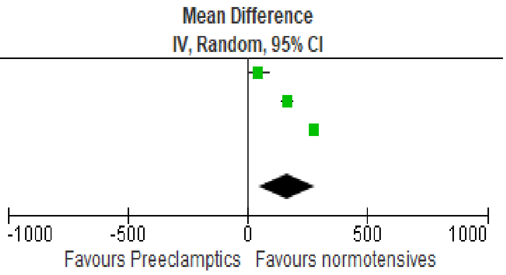

Figure 2: Forest plot showing the mean difference in UIC of normotensive and preeclamptic mothers

For peer review only

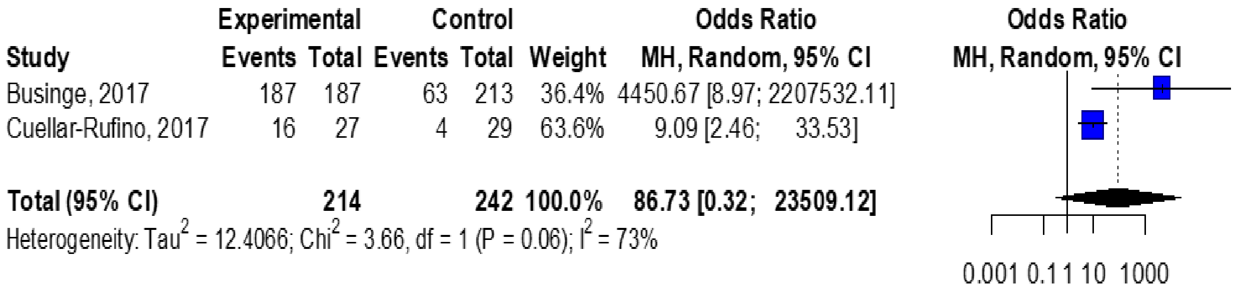

Figure 3a: Forest plot showing the odds of preeclampsia among women with UIC < 150 µg/L

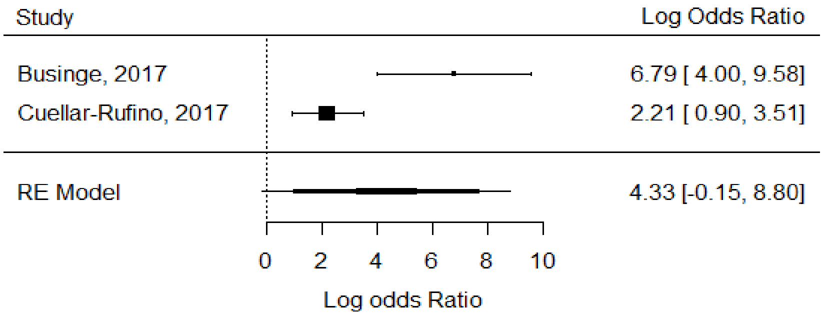

Figure 3b: Forest plot showing the Log odds of preeclampsia among women with UIC > 150 µg/L. p = 0.068, $Tau^2 = 9.8$, $I^2 = 88.94\%$.

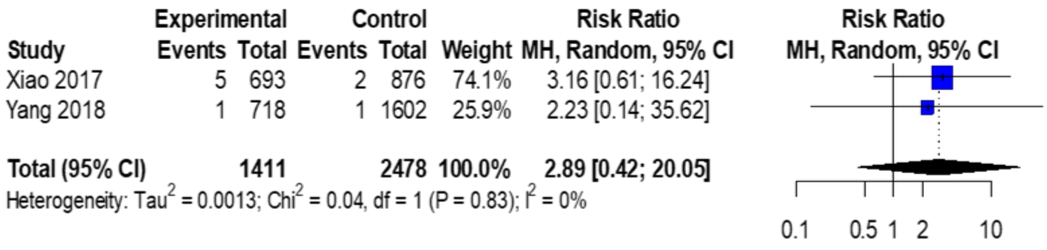

Figure 4: Forest plot showing the risk of preeclampsia among women with <150 µg/L (designated as experimental group). Pooled RR 2.89 (0.42 – 20.05), p = 0.09.

For peer review only

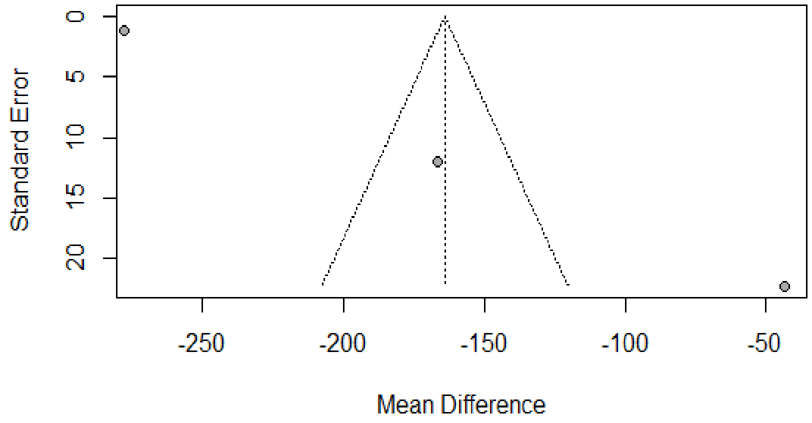

Figure 5: Funnel plot for the studies selected for the analysis of the mean difference UIC of preeclamptic women and normotensive counterparts

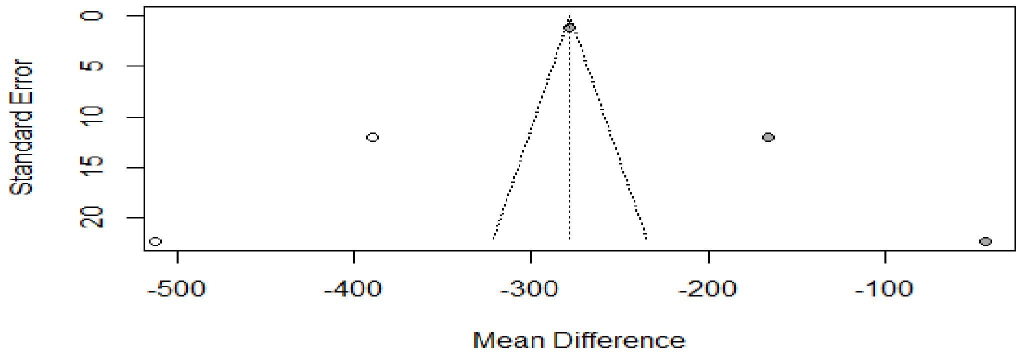

Figure 6: Funnel plots for publication bias in the studies selected for the analysis of the mean difference UIC of preeclamptic women and normotensive counterparts. The 2 imputed studies are represented by empty circles.

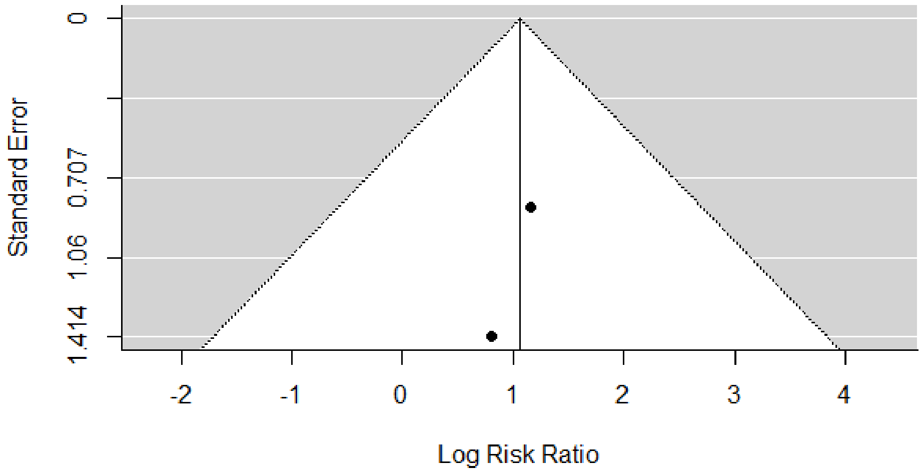

Figure 7: Funnel plot for the studies selected for the analysis of the risk of preeclampsia among women with <150 μg/L

For peer review only

PRISMA 2009 Checklist

Section/topic	#	Checklist item	Reported on page #
**TITLE**			
Title	1	Identify the report as a systematic review, meta-analysis, or both.	1
**ABSTRACT**			
Structured summary	2	Provide a structured summary including, as applicable: background; objectives; data sources; study eligibility criteria, participants, and interventions; study appraisal and synthesis methods; results; limitations; conclusions and implications of key findings; systematic review registration number.	2
**INTRODUCTION**			
Rationale	3	Describe the rationale for the review in the context of what is already known.	4
Objectives	4	Provide an explicit statement of questions being addressed with reference to participants, interventions, comparisons, outcomes, and study design (PICOS).	4
**METHODS**			
Protocol and registration	5	Indicate if a review protocol exists, if and where it can be accessed (e.g., Web address), and, if available, provide registration information including registration number.	4
Eligibility criteria	6	Specify study characteristics (e.g., PICOS, length of follow-up) and report characteristics (e.g., years considered, language, publication status) used as criteria for eligibility, giving rationale.	4
Information sources	7	Describe all information sources (e.g., databases with dates of coverage, contact with study authors to identify additional studies) in the search and date last searched.	5
Search	8	Present full electronic search strategy for at least one database, including any limits used, such that it could be repeated.	5
Study selection	9	State the process for selecting studies (i.e., screening, eligibility, included in systematic review, and, if applicable, included in the meta-analysis).	6
Data collection process	10	Describe method of data extraction from reports (e.g., piloted forms, independently, in duplicate) and any processes for obtaining and confirming data from investigators.	6
Data items	11	List and define all variables for which data were sought (e.g., PICOS, funding sources) and any assumptions and simplifications made.	6
Risk of bias in individual studies	12	Describe methods used for assessing risk of bias of individual studies (including specification of whether this was done at the study or outcome level), and how this information is to be used in any data synthesis.	6
Summary measures	13	State the principal summary measures (e.g., risk ratio, difference in means).	6
Synthesis of results	14	Describe the methods of handling data and combining results of studies, if done, including measures of consistency (e.g., I^2) for each meta-analysis.	6

For peer review only - http://bmjopen.bmj.com/site/about/guidelines.xhtml

PRISMA 2009 Checklist

Section/topic	#	Checklist item	Reported on page #
Risk of bias across studies	15	Specify any assessment of risk of bias that may affect the cumulative evidence (e.g., publication bias, selective reporting within studies).	6
Additional analyses	16	Describe methods of additional analyses (e.g., sensitivity or subgroup analyses, meta-regression), if done, indicating which were pre-specified.	6
**RESULTS**			
Study selection	17	Give numbers of studies screened, assessed for eligibility, and included in the review, with reasons for exclusions at each stage, ideally with a flow diagram.	7
Study characteristics	18	For each study, present characteristics for which data were extracted (e.g., study size, PICOS, follow-up period) and provide the citations.	7
Risk of bias within studies	19	Present data on risk of bias of each study and, if available, any outcome level assessment (see item 12).	7
Results of individual studies	20	For all outcomes considered (benefits or harms), present, for each study: (a) simple summary data for each intervention group (b) effect estimates and confidence intervals, ideally with a forest plot.	7
Synthesis of results	21	Present results of each meta-analysis done, including confidence intervals and measures of consistency.	7
Risk of bias across studies	22	Present results of any assessment of risk of bias across studies (see Item 15).	8
Additional analysis	23	Give results of additional analyses, if done (e.g., sensitivity or subgroup analyses, meta-regression [see Item 16]).	
**DISCUSSION**			
Summary of evidence	24	Summarize the main findings including the strength of evidence for each main outcome; consider their relevance to key groups (e.g., healthcare providers, users, and policy makers).	8
Limitations	25	Discuss limitations at study and outcome level (e.g., risk of bias), and at review-level (e.g., incomplete retrieval of identified research, reporting bias).	9
Conclusions	26	Provide a general interpretation of the results in the context of other evidence, and implications for future research.	9
**FUNDING**			
Funding	27	Describe sources of funding for the systematic review and other support (e.g., supply of data); role of funders for the systematic review.	9

From: Moher D, Liberati A, Tetzlaff J, Altman DG, The PRISMA Group (2009). Preferred Reporting Items for Systematic Reviews and Meta-Analyses: The PRISMA Statement. PLoS Med 6(7): e1000097. doi:10.1371/journal.pmed1000097

For more information, visit: **www.prisma-statement.org**.

For peer review only - http://bmjopen.bmj.com/site/about/guidelines.xhtml

Insufficient iodine nutrition status and the risk of preeclampsia: a systemic review and meta-analysis

Journal:	*BMJ Open*
Manuscript ID	bmjopen-2020-043505.R1
Article Type:	Original research
Date Submitted by the Author:	29-Dec-2020
Complete List of Authors:	Businge, Charles; University of Cape Town Department of Medicine, Faculty of Health Sciences; Walter Sisulu University Faculty of Health Sciences, Department of Obstetrics and Gynaecology<br>Usenbo, Anthony; Nelson Mandela Academic Hospital, Department of Anesthesiology<br>Longo-Mbenza, Benjamin; University of Kinshasa, Faculty of Medicine,; Lomo University of Research, Lomo Research Laboratory<br>Kengne , AP; South African Medical Research Council
**Primary Subject Heading**:	Obstetrics and gynaecology
Secondary Subject Heading:	Nutrition and metabolism, Epidemiology
Keywords:	Maternal medicine < OBSTETRICS, EPIDEMIOLOGY, NUTRITION & DIETETICS

I, the Submitting Author has the right to grant and does grant on behalf of all authors of the Work (as defined in the below author licence), an exclusive licence and/or a non-exclusive licence for contributions from authors who are: i) UK Crown employees; ii) where BMJ has agreed a CC-BY licence shall apply, and/or iii) in accordance with the terms applicable for US Federal Government officers or employees acting as part of their official duties; on a worldwide, perpetual, irrevocable, royalty-free basis to BMJ Publishing Group Ltd ("BMJ") its licensees and where the relevant Journal is co-owned by BMJ to the co-owners of the Journal, to publish the Work in this journal and any other BMJ products and to exploit all rights, as set out in our licence.

The Submitting Author accepts and understands that any supply made under these terms is made by BMJ to the Submitting Author unless you are acting as an employee on behalf of your employer or a postgraduate student of an affiliated institution which is paying any applicable article publishing charge ("APC") for Open Access articles. Where the Submitting Author wishes to make the Work available on an Open Access basis (and intends to pay the relevant APC), the terms of reuse of such Open Access shall be governed by a Creative Commons licence – details of these licences and which Creative Commons licence will apply to this Work are set out in our licence referred to above.

Other than as permitted in any relevant BMJ Author's Self Archiving Policies, I confirm this Work has not been accepted for publication elsewhere, is not being considered for publication elsewhere and does not duplicate material already published. I confirm all authors consent to publication of this Work and authorise the granting of this licence.

For peer review only

Insufficient iodine nutrition status and the risk of preeclampsia: a systemic review and meta-analysis

*Charles Bitamazire Businge[1, 2], Anthony Usenbo[3], Benjamin Longo-Mbenza[4], Andre Pascal Kengne[2, 5]

[1]Department of Obstetrics and Gynaecology, Faculty of Health Sciences, Walter Sisulu University, Private Bag x1 WSU, 5117, Mthatha, South Africa

[2]Department of Medicine, Faculty of Health Sciences, University of Cape Town, Cape Town, South Africa

Email: bsnbit001@myuct.ac.za /cbusingae@gmail.com /cbusinge@wsu.ac.za

[3]Nelson Mandela Academic Hospital, Mthatha, Eastern Cape, South Africa.

Email: tonyusenbo@gmail.com

[4]Faculty of Medicine, University of Kinshasa, Democratic Republic of Congo and LOMO University of Research, Kinshasa, Democratic Republic of Congo

Email: longombenza@gmail.com / longombenza@yahoo.fr

[5]Non-Communicable Disease Research Unit, South African Medical Research Council, Cape Town, South Africa
Email: Andre.Kengne@mrc.ac.za

*Corresponding author

Word count - 4207

For peer review only

Abstract

Background: Although sub-clinical hypothyroidism (SCH) in pregnancy is one of the established risk factors for preeclampsia, the link between iodine deficiency, the main cause of hypothyroidism, and preeclampsia remains uncertain. We conducted a systematic review to determine the iodine nutrition status of pregnant women with and without preeclampsia and the risk of preeclampsia due to iodine deficiency.

Methods: MEDLINE, EMBASE, Google Scholar, SCOPUS and Africa Wide Information were searched up to June 30th 2020. Random-effects models meta-analysis was used to pool mean difference UIC between preeclamptic and normotensive controls; and pool odds ratios and incidence rates of preeclampsia among women with UIC <150 µg/L.

Results: Five eligible studies were included in the meta-analysis. There was a significant difference in the pooled mean UIC of 254 preeclamptic women and 210 normotensive controls enrolled in three eligible case-control studies (mean UIC 164.4 µg/L [95% CI 45.1 – 283.6, p < 0.01, I^2 >50]). The overall proportions of preeclampsia among women with UIC <150 µg/L and UIC >150 µg/L in two cross-sectional studies were 203/214 and 67/247 respectively with a pooled odds ratio of 0.01 (95% CI 0.00 – 4.23, p = 0.14, I^2 >50) for preeclampsia among women with UIC >150 µg/L. The overall incidence of preeclampsia among women with UIC <150 µg/L and UIC >150 µg/L in two cohort studies were 6/1411 and 3/2478 respectively with a pooled risk ratio of 2.85 (95% CI 0.42 – 20.05, p = 0.09, I^2<25)

Conclusion: Although preeclamptic women seem to have lower UIC than normotensive pregnant women, the available data is insufficient to provide a conclusive answer on association of iodine deficiency with preeclampsia risk.

Key words

Iodine deficiency, pregnancy, preeclampsia

Strengths and limitations of this study

- The current study is among the first systematic reviews that have ascertained the relationship between insufficient iodine nutrition status and pre-eclampsia.
- This review was however been limited by the small number of eligible studies coupled with small sample sizes.

For peer review only - http://bmjopen.bmj.com/site/about/guidelines.xhtml

For peer review only

- The varied study designs coupled with a considerable degree of heterogeneity precluded the pooling of all the results.

This review is registered with PROSPERO (registration no. CRD42018099427).

For peer review only

For peer review only - http://bmjopen.bmj.com/site/about/guidelines.xhtml

INTRODUCTION

Sub-clinical hypothyroidism (SCH) is a risk factor for preeclampsia, which is a prominent cause of maternal and perinatal morbidity and mortality. [1-3] Iodine deficiency, which is exacerbated by pregnancy related physiological changes, is a leading cause of hypothyroidism.[4-5] Hence, among women within the reproductive age bracket, insufficient nutrition status prior to the onset of pregnancy, which potentially worsens during the course of pregnancy, could increase the risk of preeclampsia like is the case for foetal neurological complications, particularly in endemic iodine deficiency settings. [6 7]

Over two billion people live in areas with iodine insufficiency.[8] Iodine deficiency is on the rise in areas originally thought to be iodine sufficient, despite concerted worldwide efforts to promote iodine fortification. This is partly attributed to high concentration of perchlorate and thiocyanate in water sources and the diet, which impairs the uptake of iodine by the thyroid gland, particularly among individuals with TSH-related thyroid stimulation secondary to iodine deficiency; and to ineffective implementation and monitoring of dairy and bread based iodine supplementation strategies. [9-12]

In this systematic review and meta-analysis, we sought to establish if there a difference in the urinary iodine concentration (UIC) of pregnant women with and without pre-eclampsia; and whether pregnant women with insufficient iodine nutrition status are at increased risk of pre-eclampsia.

The study is reported according to the Preferred Reporting Items for Systematic reviews and Meta-Analysis (PRISMA) Guidelines, [13] and was based on a protocol that was registered with the International Prospective Register of Systematic Reviews, PROSPERO (registration no. CRD42018099427).

Methods

Eligibility criteria

Inclusion criteria

The selection of studies for inclusion was guided by the Population, Intervention/exposure, Comparison and Outcome (PICO) protocol. The target population were pregnant women and the exposure was insufficient iodine nutrition status before pregnancy for cohort studies, and insufficient iodine nutrition status during pregnancy for case-control studies. The iodine nutrition status was defined according to the World Health Organization / International Council

For peer review only - http://bmjopen.bmj.com/site/about/guidelines.xhtml

for Control of Iodine Deficiency Disorders (ICCIDD) classification of iodine intake using median urinary iodine concentration (UIC).[14 15] For pregnant women, a urine iodine concentration (UIC) <150, 150-249, 250-499 and >500µg µg/L is considered an estimate of insufficient, adequate, more than adequate and excessive iodine nutritional status, respectively.[15] The comparators were study participants with sufficient iodine nutrition status (UIC ≥150 µg/L) during pregnancy. [14 15] The outcomes were the prevalence and incidence rates of preeclampsia among women with and without adequate iodine nutrition status from which the odds ratios for case control and risk ratios for cohort studies were determined.

Preeclampsia was defined as new onset hypertension after 20 weeks of amenorrhoea characterised by elevated systolic blood pressure (SBP ≥140 mmHg) and/or diastolic blood pressure (DBP ≥90 mmHg), based on two measurements four hours apart; or SBP>160 mmHg and/or DBP>110 mmHg from a single measurement. Elevated BP had to be accompanied by at least one of the following: proteinuria in 24 h-urine ≥ 300 mg or protein/creatinine ratio ≥ 0.3mg/mg or urine protein measured by dipstick ≥ 2+; thrombocytopenia (platelets count less than 150,000/µL), kidney insufficiency (serum creatinine levels above 90 µmol/L), decreased liver function (AST and ALT twice higher than the upper limit of the reference interval), compromised lung function or pulmonary oedema, visual or other symptoms and signs of impaired cerebral function.[16] There may be considerable heterogeneity if preeclampsia has been variably defined in different studies that are eligible for inclusion in the current systematic review.

Exclusion criteria

Studies were excluded if they lacked means, medians, odds ratios, incidence and prevalence rates data to compute them even after repeated unsuccessful attempts to contact the authors via email for relevant information. Letters to editors, reviews, commentaries, editorials and any publication without primary data were also excluded.

Patient and public involvement

There was no involvement of the public or patients.

Search Strategy and Selection Criteria

We searched PubMed, Scopus, Web of Science, Academic Search Premier, Africa Wide Information, CINAHL, Cochrane Library, Google Scholar, and Health Source: Nursing/Academic Edition databases for all published studies on iodine deficiency and

For peer review only - http://bmjopen.bmj.com/site/about/guidelines.xhtml

preeclampsia up to 30th June 2020. This search was conducted using a predefined comprehensive and sensitive search strategy (Table 1) combining relevant terms and synonyms which are variably used to denote abnormally high blood pressure in pregnancy and insufficient iodine intake or iodine deficiency as detailed in the published protocol for this review.[17]

Study Selection and Data Extraction

Two authors (CBB and AU) independently screened the titles and abstracts of identified studies. Citations and abstracts were initially screened and duplicate citations excluded. Titles and abstracts were then screened following inclusion criteria described in the protocol[17], after which the full-texts of potentially eligible articles were obtained. These full texts were screened using a standardised and pretested form to include eligible studies. Disagreements were resolved by consensus. For each study, one reviewer (CBB) extracted the data and a second reviewer (AU) checked the accuracy. For the 5 studies included here, there were no disagreements between the two reviewers. Figure 1 shows the flowchart for the selection process. The following data were extracted from the eligible studies: studies characteristics (authors, years, design, and study regions), study population (age, and sample size), iodine nutrition status of the various study groups, and the methods of outcomes measurement.

Quality Assessment

Two reviewers (CBB and AU) independently scored the risk of bias and the quality of included studies (Table 2a and Table 2b) using the Newcastle-Ottawa scale. [18] Inter-rater agreement on screening, data abstraction and methodological quality (selection, comparability of groups and ascertainment of exposure/outcome) was assessed using Cohen's κ coefficient. [19] The Kappa value for interrater agreement for quality assessment was 0.694 ($p < 0.001$). Discrepancies were resolved by consensus.

Data synthesis, analysis and assessment of heterogeneity

The analysis was performed with the Review Manager (RevMan) Software, version 5.4 (The Nordic Cochrane Centre, The Cochrane Collaboration) and the 'meta' and 'metafor' packages of the statistical software R (version 4.0.2, The R Foundation for statistical computing, Vienna, Austria). For the outcomes of interest (means, prevalence and incidence rates), random effects model meta-analyses were used to pool estimates across studies with similar design. [20] The

For peer review only - http://bmjopen.bmj.com/site/about/guidelines.xhtml

degree of heterogeneity across studies was assessed using the Cochrane Q statistic and inconsistency index (I^2) statistic and values ranked as indicating low: I^2<25%; moderate: 25%–50%; high heterogeneity: I^2>50%).[21] The Egger funnel plot was used to check for publication bias.[22]

Results

The review process

The process for selecting the relevant studies is summarised in Figure 1. In total, 2380 records were identified via database searches. After removing duplicates, we scanned the titles and abstracts of 522 articles, of which 11 full texts were further reviewed. Of these, 5 articles met criteria for inclusion in the current systematic review.

Characteristics of included studies

All the 5 included studies were categorised as having a low risk of bias. Their characteristics are summarised in Table 3. Three were institutional-based case-control studies one from each of the following countries: Turkey, Mexico and the Democratic Republic of Congo while 2 were prospective cohort studies were from two different provinces (Henan and Liaoning) in China.[23 - 28]

Meta-analysis

Mean difference in urinary iodine concentration of preeclamptic and normotensive women

Three studies reported the mean UIC of preeclamptic and normotensive pregnant women.[23– 25] Overall there was a significant and positive mean difference in UIC and standardised mean UIC of normotensive pregnant women and preeclamptic women, with substantial heterogeneity across studies (Fig 2).

The risk of preeclampsia among women with UIC < 150 µg/L

Two case-control studies had data with proportions of preeclamptic and normotensive participants with UIC above or below <150 µg/L.[22 23] The odds of preeclampsia among women with UIC<150 µg/L was above unity for individual studies but the pooled odds ratio of 86.73 (0.32, 23509.12) was not significant with substantial heterogeneity across studies (I^2 = 73%) (Fig 3a and 3b).

For peer review only - http://bmjopen.bmj.com/site/about/guidelines.xhtml

The incidence of preeclampsia in the two cohort studies was 2/2320 and 7/1576 respectively.[26 27] There was no difference in the incidence of preeclampsia among participants with or without low UIC (UIC <150μg/L) as shown in Figure 4.

Publication Bias

Visual inspection of funnel plot symmetry suggested potential publication bias for the studies included in the meta-analysis of UIC difference preeclamptic and normotensive counterparts as well as the odds of preeclampsia among women with UIC <150 μg/L (Figure 5).

After adjustment of the effect size for potential publication bias using the trim-and-fill correction, 2 potentially missing studies (Figure 6) were imputed in funnel plot (Mean UIC differences of -389.60 [-413.02; -366.17] and -512.50 [-556.23; -468.78] respectively. With potential inclusion of the missing studies the pooled mean UIC was estimated to be -278.0000 [-438.3025; -117.6975] which is significantly different from the pooled estimate of the 3 included studies (p <0.001).

The funnel plot for the cohort studies included in the assessment of the incidence of preeclampsia among women with UIC <150 μg/L was not suggestive of potential publication bias (Figure 7).

Discussion

The current review has shown that preeclamptic women have significantly lower mean UIC than their normotensive counterparts. This trend was observed in all the 3 included studies despite being from 3 different continents: Africa, Europe and South America.[23 - 25] This association between low UIC and preeclampsia may reflect inadequate iodine intake predating pregnancy persisting till the third trimester that may increase the risk of preeclampsia among susceptible women. A recent Norwegian study reported that among women with mild to moderate deficiency, long-term preconception iodine supplementation was associated with reduced incidence of preeclampsia.[7]

Although there was a trend towards a positive association between low UIC (UIC<150μg/L) in the third trimester and preeclampsia for the included case-control and cohort studies, the pooled odds and risk ratios showed a non-significant association. The small number of eligible

For peer review only - http://bmjopen.bmj.com/site/about/guidelines.xhtml

studies which also had substantially high heterogeneity may partially account for this result. Hence the available data is insufficient to provide a definitive answer on the risk of preeclampsia associated with low UIC in the third trimester.

Iodine deficiency is thought to predispose to incident preeclampsia through two mechanisms. The first one is the reduction of the anti-oxidant capacity of the placenta, which is one of the organs where the sodium iodine symporter maintains a high concentration of iodine which among other roles, is thought to reduce oxidative stress and lipid peroxide formation which are elevated in patients with preeclampsia.[28 – 30] The second mechanism is persistent iodine deficiency predisposing to elevated TSH. TSH operating via its endothelial receptors has been shown to diminish endothelial Nitric Oxide and prostacyclin production as well as upregulate endothelin production, which lead to endothelial dysfunction and systemic vasoconstriction.[31-33]. Since baseline pre-pregnancy as well as serial pregnancy UIC analyses were not carried out in the two cohort studies, it remains uncertain whether the iodine nutritional status at enrolment truly reflected the iodine nutritional status before pregnancy and for the remaining duration of the pregnancy following enrolment. Iodine nutritional status is likely to change with dietary habits and the progressive physiological changes of pregnancy. This could lead to misclassification of study participants, and dilute the association between iodine deficiency and preeclampsia.[34] The estimation of maternal intra-thyroid iodine concentration, even though more technical, has been proposed as a more objective measure of pre-pregnancy iodine nutrition status than spot UIC. [35] Concurrent measurement of spot UIC and serum thyroglobulin may help identify individuals with long-term exposure to iodine deficiency in studies where it is not possible to measure serial UIC and intra-thyroid iodine concentration.[36 37]

Limitations

This review was limited by the small number of eligible studies with small sample sizes and substantial degree of heterogeneity. The varied research designs of the eligible studies precluded the pooling of all the test results.

Conclusion

Although the UIC of women who present with preeclampsia seems to be lower than that of women who remain normotensive till delivery, the available data is insufficient to reliably draw a conclusion on the association of iodine deficiency with the risk of preeclampsia. More well-designed and adequately powered studies that also include the estimation of pre-pregnancy iodine nutrition status are needed to address this question.

For peer review only - http://bmjopen.bmj.com/site/about/guidelines.xhtml

Funding

This study is part of a research project supported by the Discovery Foundation Rural Fellowship Grant number 038372. APK is employed by the South African Medical Research Council.

Authors' contribution

CBB and APK conceived and designed the study. CBB carried out the literature search. CBB and AU screened the identified studies and extracted data from eligible studies. CBB was responsible for data analysis and writing the first manuscript. APK, AU and LMB took part in critical revision of the first manuscript. All the authors read and approved the final version of the manuscript.

Competing interests

There are no competing interests.

Patient consent for publication

This not applicable since the current study is a systematic review and meta-analysis of already published data.

Data sharing

All data relevant to the study are included in the article

Figure captions

Figure 1: Study selection flowchart

Figure 2: Forest plot showing the mean difference in UIC of normotensive and preeclamptic mothers

Figure 3a: Forest plot showing the odds of preeclampsia among women with UIC < 150 µg/L

Figure 3b: Forest plot showing the Log odds of preeclampsia among women with UIC > 150 µg/L. p = 0.068, $Tau^2 = 9.8$, $I^2 = 88.94\%$.

Figure 4: Forest plot showing the risk of preeclampsia among women with <150 µg/L (designated as experimental group). Pooled RR 2.89 (0.42 – 20.05), p = 0.09.

Figure 5: Funnel plot for the studies selected for the analysis of the mean difference UIC of preeclamptic women and normotensive counterparts

For peer review only - http://bmjopen.bmj.com/site/about/guidelines.xhtml

Figure 6: Funnel plots for publication bias in the studies selected for the analysis of the mean difference UIC of preeclamptic women and normotensive counterparts. The 2 imputed studies are represented by empty circles.

Figure 7: Funnel plot for the studies selected for the analysis of the risk of preeclampsia among women with <150 μg/L.

References

1. Abalovich M, Gutierrez S, Alcaraz G, *et al*. Overt and subclinical hypothyroidism complicating pregnancy. *Thyroid* 2002;12(1): 63-68.

2. Khanam M, Ilias M. Study of Thyroid Hormonal Status in Preeclamptic Patients. *Medicine Today* 2013;25(2): 63 - 66.

3. Wilson KL, Casey BM, McIntire DD, *et al*. Subclinical thyroid disease and the incidence of hypertension in pregnancy. *Obstet Gynecol*, 2012;119(2 Pt 1):315-20.

4. Lazarus JH. Screening for Thyroid function in Pregnancy: Is It worthwhile? Journal of thyroid Research. [Online] 2011.Available: http://.dx.doi.org/10.4061/2011/397012.

5. Jameson LJ, Mandel SJ, Weetman AP. Ch 405- Disorders of the Thyroid gland. In: Kasper DL, Hauser SL, Jameson JL, Fauci AS, Longo DL, Loscalzo J, editors. Principles of Internal Medicine 19th Ed. New York: McGraw Hill Education, 2015; p 2283 - 2308.

6. Pharoah P, Buttfield IH, Hetzel BS. Neurological damage to the fetus resulting from severe iodine deficiency during pregnancy. Int J Epidemiol 2012;41(3):589-92.

7. Abel MH, Caspersen IH, Sengpiel V, Jacobsson B, Meltzer HM, Magnus P, Alexander J, Brantsæter AL. Insufficient maternal iodine intake is associated with subfecundity, reduced foetal growth, and adverse pregnancy outcomes in the Norwegian Mother, Father and Child Cohort Study. BMC Med 2020;18(1):211.

8. Andersson M, Karumbunathan V, Zimmermann MB. Global iodine status in 2011 and trends over the past decade. *Journal of Nutrition* 2012;142(4):744-750.

9. Zimmermann MB. Iodine deficiency in industrialized countries. *Clinical Endocrinology* 2011;75: 287-288.

For peer review only

10. De Groef, B, Decallonne BR, Van der Geyten S, *et al*. Perchlorate versus other environmental sodium/iodide symporter inhibitors: potential thyroid related health effects. *Eur J Endocrinol* 2006;155(1):17-25.

11. Steinmaus C, Miller MD. Howd R. Impact of smoking and thiocyanate on perchlorate and thyroid hormone associations in the 2001-2002 national health and nutrition examination survey. *Environ Health Perspect* 2007;115(9):1333-8.

12. Vidal, ZEO, Rufino SC, Tlaxcalteco EH, *et al*. Oxidative Stress Increased in Pregnant Women with Iodine Deficiency. *Biol Trace Elem Res* 2014;157: 211.

13. Moher, D., Shamseer, L., Clarke, M., et al. Preferred reporting items for systematic review and meta-analysis protocols (PRISMA-P) 2015 statement. Syst Rev 2015;4:1.

14. WHO, UN Children's Fund, International Council for the Control of Iodine Deficiency Disorders. Assessment of iodine deficiency disorders and monitoring their elimination. A guide for programme managers, 3rd edn. Geneva: World Health Organization, 2007.

15. WHO (World Health Organization). Proceedings of the WHO Technical Consultation on control of iodine deficiency in pregnant women and young children. Geneva: WHO, 2005.

16. Tranquilli AL, Dekker G, Magee L, Roberts J, Sibai BM, Steyn W, Zeeman GG, Brown MA. The classification, diagnosis and management of the hypertensive disorders of pregnancy: a revised statement from the ISSHP. Pregnancy Hypertens 2014;4:97–104.

17. Businge CB, Madini N, Longo-Mbenza B, Kengne AP. Insufficient iodine nutrition status and the risk of pre-eclampsia: a protocol for systematic review and meta-analysis. BMJ Open. 2019 May;9(5):e025573. DOI: 10.1136/bmjopen-2018-025573.

18. Wells GA, Shea B, O'Connell D, Peterson J, Welch V, Losos M, et al. The Newcastle-Ottawa Scale (NOS) for assessing the quality of nonrandomised studies in meta-analyses [cited 2018 Oct 06]. Available from: http://www.evidencebasedpublichealth.de/download/Newcastle_Ottawa.

19. McHugh ML. Interrater reliability: the kappa statistic. Biochem Med (Zagreb), 2012;22(3): 276–282.

20. Riley RD, Higgins JPT, Deeks JJ. Interpretation of random effects meta-analyses. BMJ 2011;342:d549.

21. Higgins JP, Thompson SG, Deeks JJ, et al. Measuring inconsistency in meta-analyses. BMJ 2003;327:557-60.

For peer review only - http://bmjopen.bmj.com/site/about/guidelines.xhtml

22. Egger M, Davey SG, Schneider M, Minder C. Bias in meta-analysis detected by a simple, graphical test. BMJ 1997;315:629.

23. Gulaboglu M, Borekci B, Delibas I. Urine iodine levels in preeclamptic and normal pregnant women. Biol Trace Elem Res 2010;136:249–257.

24. Cuellar-Rufino S, Navarro-Meza M, García-Solís P, Xochihua-Rosas I, Arroyo-Helguera O. Iodine levels are associated with oxidative stress and antioxidant status in pregnant women with hypertensive disease. Nutr Hosp. 2017 Jun 5;34(3):661-666.

25. Businge BC, Longo-Mbenza B, Adeniyi OV, Nzuzi Babeki V, Kitambala Kaboka A, Mvitu Muaka M, Nkanga Nganga SM, Tsobo CM, Vangu R, Diambu EM and l Ter Goon D. Iodine Deficiency in Pregnancy as a Predictor of Sub-clinical Hypothyroidism, Preeclampsia and Future Cardiovascular Disease. J Clin Nutri 2017; 9:118-123.

26. Yang J, Liua Y, Liub H, Zhenga H, Lia X, Zhua L, WangA Z. Associations of maternal iodine status and thyroid function with adverse pregnancy outcomes in Henan Province of China. J Trace Elements Med Biol 2018; 47:104–110.

27. Xiao Y, Sun H, Li C, Li Y, Peng S, Fan C, Teng W, Shan Z. Effect of Iodine Nutrition on Pregnancy Outcomes in an Iodine-Sufficient Area in China. Biol Trace Elem Res 2018; 182:231–237.

28. Burns R, O'Herlihy C, Smyth PP. The placenta as a compensatory iodine storage organ. Thyroid 2011;21(5):541-6.

29. Vidal ZEO, Rufino SC, Tlaxcalteco EH, et al. Oxidative Stress Increased in Pregnant Women with Iodine Deficiency. Biol Trace Elem Res 2014;157: 211.

30. Brown SHJ, Eather SR, Freeman DJ, Meyer BJ, Mitchell TW. A Lipidomic Analysis of Placenta in Preeclampsia: Evidence for Lipid Storage. PLoS ONE 2016; 11(9): e0163972. doi:10.1371/journal.pone.0163972.

31. Tian, L., Zhang, L., Liu, J. Guo, T., Gao, C. and Ni1, J. Effects of TSH on the function of human umbilical vein endothelial cells. Journal of Molecular Endocrinology 2014; 52: 215-222.

32. Dardano, A., Ghiadoni, L., Plantinga, Y., Caraccio, N., Bemi, A., Duranti, E., Taddei, S., Ferrannini, E., Salvetti, A. and Monzani, F. Recombinant Human Thyrotropin Reduces Endothelium-Dependent Vasodilation in Patients Monitored for Differentiated Thyroid Carcinoma. J Clin Endocrinol Metab 2006; 91: 4175-4178.

33. Lioudaki, E., Mavroeidi, N.G., Mikhailidis, D.P., Emmanouel S. Ganotakis, E.S. Subclinical hypothyroidism and vascular risk: An update. Hormones 2013; 12(4):495-506.

For peer review only - http://bmjopen.bmj.com/site/about/guidelines.xhtml

34. Haine D, Dohoo I, Dufour S. Selection and Misclassification Biases in Longitudinal Front. Vet. Sci. 2018; 5:99. doi: 10.3389/fvets.2018.00099.

35. Dineva M, Fishpool H, Rayman MP, Mendis J, Bath SC. Systematic review and meta-analysis of the effects of iodine supplementation on thyroid function and child neurodevelopment in mildly-to-moderately iodine-deficient pregnant women. American Journal of Clinical Nutrition 2020; 112(2):389–412.

36. Du Y, Gao YH, Feng ZY, Meng FG, Fan LJ, Sun DJ. Serum Thyroglobulin—A Sensitive Biomarker of Iodine Nutrition Status and Affected by Thyroid Abnormalities and Disease in Adult Populations. Biomed Environ Sci, 2017; 30(7): 508-516.

37. Bath SC, Pop VJM, Furmidge-Owen VL, Broeren MAC, Rayman MP.Thyroglobulin as a Functional Biomarker of Iodine Status in a Cohort Study of Pregnant Women in the United Kingdom. Thyroid 2017; 27(3):426-433.

Table 1 Search strategy for MEDLINE [15]

**Population: Pregnant women with Pre-eclampsia**		
#1	MeSH terms	Pregnant Women [Mesh] OR Pregnancy [Mesh] OR Pregnancy Trimesters [Mesh]
#2	Free text	Pregnancy OR Pregnant women OR expectant mothers
#3	#1 OR #2	
#4	MeSH terms	Pre-Eclampsia [Mesh] OR Eclampsia [Mesh] OR Hypertension [Mesh]
#5	Free text	Preeclampsia OR Pre-eclampsia OR Eclampsia OR Hypertension OR Hypertensive OR High blood pressure
#6	#4 OR #5	
**Exposure: Iodine deficiency**		
#7	MeSH terms	Iodine [Mesh]
#8	Free text	Iodine
#9	#7 OR #8	
#10	#3 AND #6 AND #9	

Table 2a: Risk of bias assessment (Reviewer CBB)

For peer review only - http://bmjopen.bmj.com/site/about/guidelines.xhtml

Study	Study type	Selection	Comparability	Outcome/exposure	≠AHRQ scale (good/fair/poor)
**Gulaboglu, 2009**	**Case control**	***	**	***	**Good**
**Cuellar-Rufino, 2017**	**Case control**	****	**	***	**Good**
**Businge, 2017**	**Case control**	****	**	**	**Good**
**Yang, 2018**	**Cohort**	****	**	***	**Good**
**Xiao, 2017**	**Cohort**	****	**	***	**Good**

≠AHQR = Agency for Healthcare Research and Quality

Table 2b: Risk of bias assessment (Reviewer AU)

Study	Study Type	Selection	Comparability	Outcome/ Exposure	≠AHRQ scale (good/fair/poor)
**Gulaboglu 2009**	Case-control	***	**	***	Good
**Cuellar-Rufino, 2017**	Case-control	***	**	**	Good
**Businge 2017**	Case-control	****	**	***	Good
**Yang 2018**	Cohort study	****	**	***	Good
**Xiao 2017**	Cohort study	****	**	***	Good

≠AHQR = Agency for Healthcare Research and Quality

For peer review only - http://bmjopen.bmj.com/site/about/guidelines.xhtml

443 Table 3: Characteristics of included studies

First author, year	Country	Study design	Study period	Cases(n)	Controls(n)	Comparator	Diagnostic criteria
**Gulaboglu, 2009**	Turkey	Case-control	Not stated	Severe preeclampsia (40)	Normotensives (18)	Mean IUC	Sandell–Kolthoff reaction
**Cuellar-Rufino, 2017**	Mexico	Case-control	Jan – April 2015	Preeclampsia (20)	Normotensives (37)	• Mean IUC<br>• IUC<150 µg/L	Fast colorimetric method
**Businge, 2017**	Congo Democratic Republic	Case-control	Jan 2007 to December 2008	Preeclampsia (68) and Severe preeclampsia/ Eclampsia (182)	Normotensives (150)	• Mean IUC<br>• IUC<150 µg/L	Sandell–Kolthoff reaction
**Yang, 2018**	Henan Province, China	cohort	July to September 2015	• Incident preeclampsia 01/718 for women with IUC <150 µg/L<br>• Incident gestational HT 17/718 for women with IUC <150 µg/L	• Incident preeclampsia 01/1602 for women with IUC >150 µg/L<br>• Incident gestational HT 25/1602 for women with IUC >150 µg/L	• IUC<150 µg/L	Fast colorimetric method
**Xiao, 2017**	Liaoning province, China	Cohort	2012 - 2014	• Incident preeclampsia 05/693 for women with IUC <150 µg/L<br>• Incident gestational HT 18/693 for women with IUC <150 µg/L	• Incident preeclampsia 02/876 for women with IUC >150 µg/L<br>• Incident gestational HT 25/876 for women with IUC >150 µg/L	• IUC<150 µg/L	Sandell–Kolthoff reaction

444

445

For peer review only - http://bmjopen.bmj.com/site/about/guidelines.xhtml

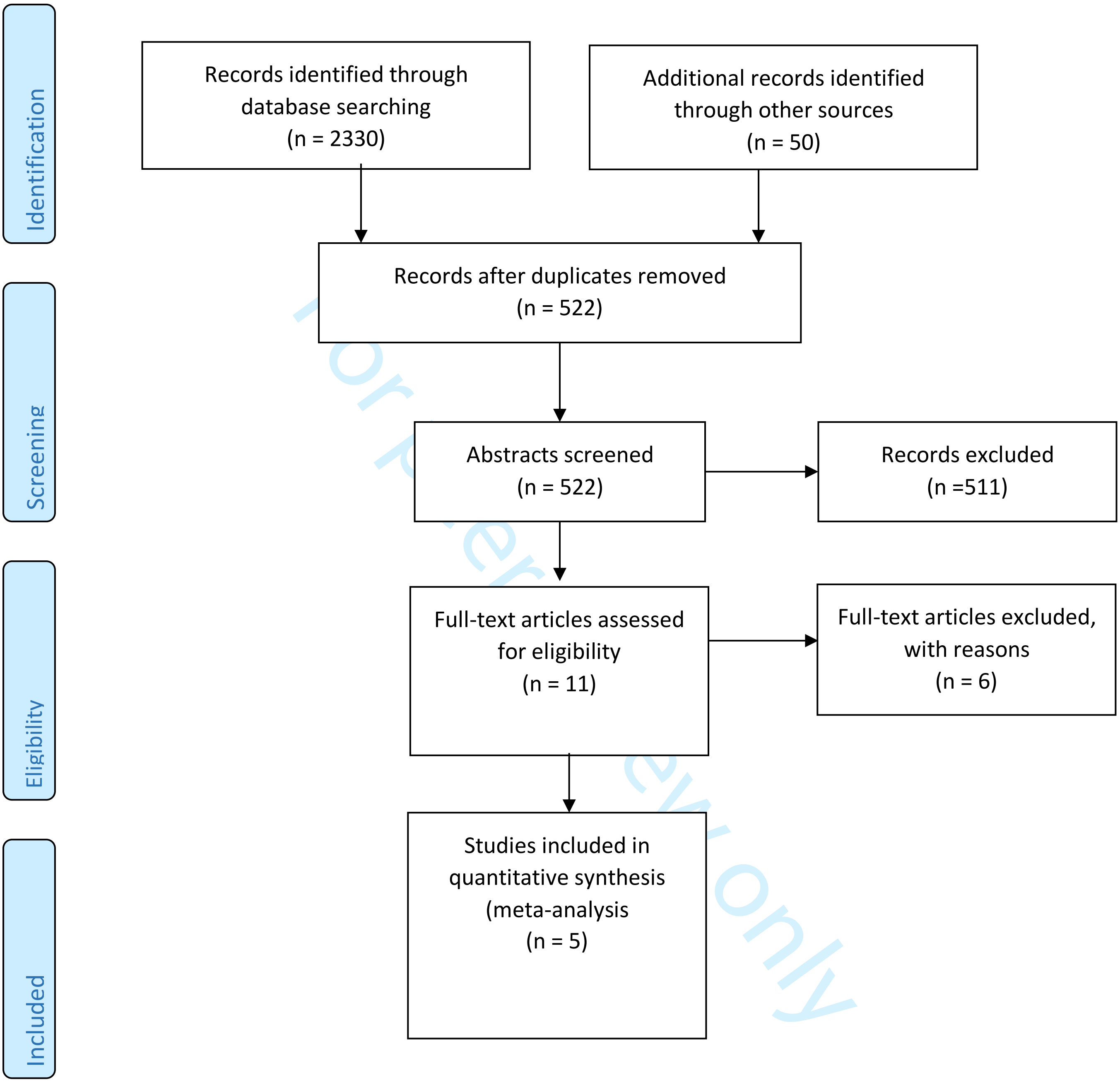

Figure 1: Study selection flowchart

For peer review only - http://bmjopen.bmj.com/site/about/guidelines.xhtml

Study or Subgroup	Normotensive controls Mean	SD	Total	Preeclamptics Mean	SD	Total	Weight	Mean Difference IV, Random, 95% CI
Cuellar-Rufino 2017	185.7	77.2	20	142.2	84.8	37	32.5%	43.50 [0.01, 86.99]
Gulaboglu 2009	208.9	64	40	42.5	27	18	33.5%	166.40 [142.97, 189.83]
Businge 2017	423.9	13	150	145.9	7.5	200	34.0%	278.00 [275.67, 280.33]
**Total (95% CI)**			**210**			**255**	**100.0%**	**164.36 [45.14, 283.58]**

Heterogeneity: Tau² = 10891.98; Chi² = 196.65, df = 2 (P < 0.00001); I² = 99%
Test for overall effect: Z = 2.70 (P = 0.007)

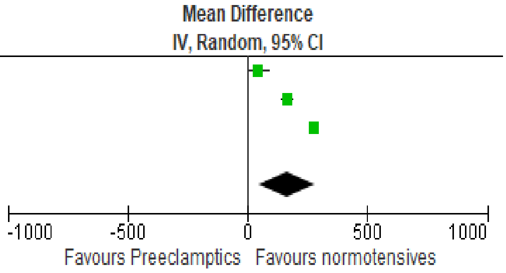

Figure 2: Forest plot showing the mean difference in UIC of normotensive and preeclamptic mothers

For peer review only

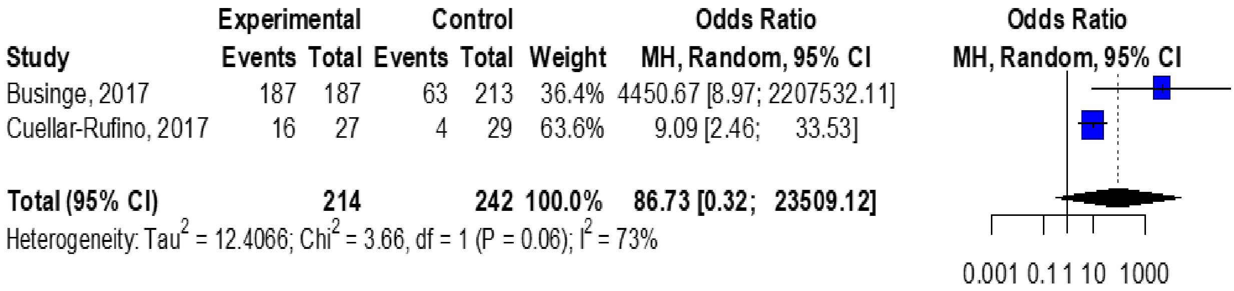

Figure 3a: Forest plot showing the odds of preeclampsia among women with UIC < 150 µg/L

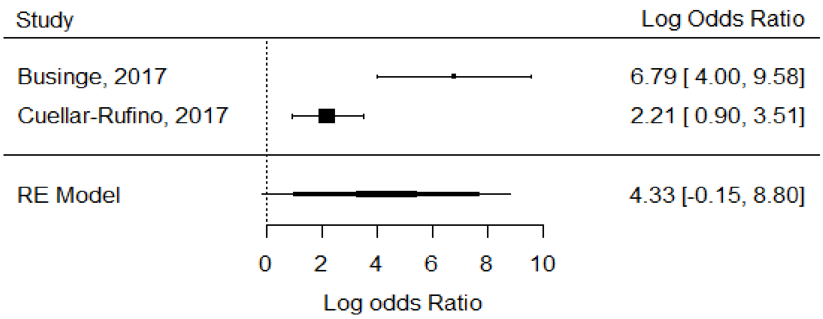

Figure 3b: Forest plot showing the Log odds of preeclampsia among women with UIC > 150 µg/L. p = 0.068, $Tau^2 = 9.8$, $I^2 = 88.94\%$.

Study	Experimental Events	Experimental Total	Control Events	Control Total	Weight	Risk Ratio MH, Random, 95% CI
Xiao 2017	5	693	2	876	74.1%	3.16 [0.61; 16.24]
Yang 2018	1	718	1	1602	25.9%	2.23 [0.14; 35.62]
**Total (95% CI)**		**1411**		**2478**	**100.0%**	**2.89 [0.42; 20.05]**

Heterogeneity: $Tau^2 = 0.0013$; $Chi^2 = 0.04$, df = 1 (P = 0.83); $I^2 = 0\%$

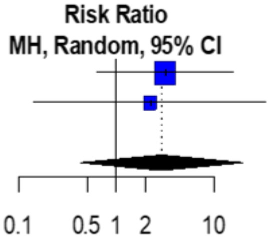

Figure 4: Forest plot showing the risk of preeclampsia among women with <150 µg/L (designated as experimental group). Pooled RR 2.89 (0.42 – 20.05), p = 0.09.

For peer review only

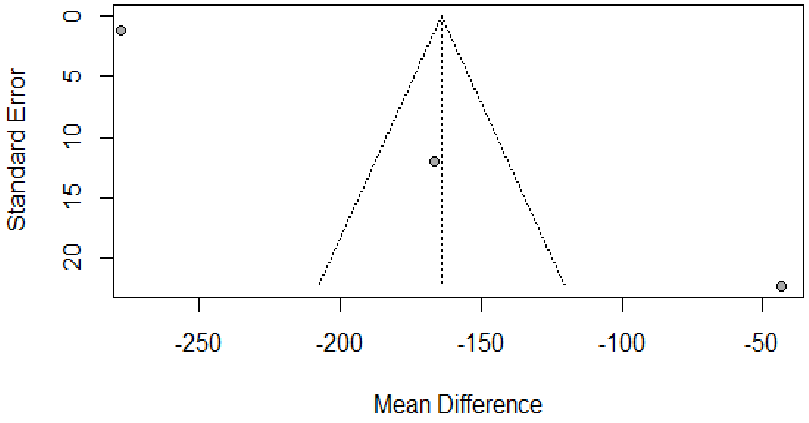

Figure 5: Funnel plot for the studies selected for the analysis of the mean difference UIC of preeclamptic women and normotensive counterparts

For peer review only

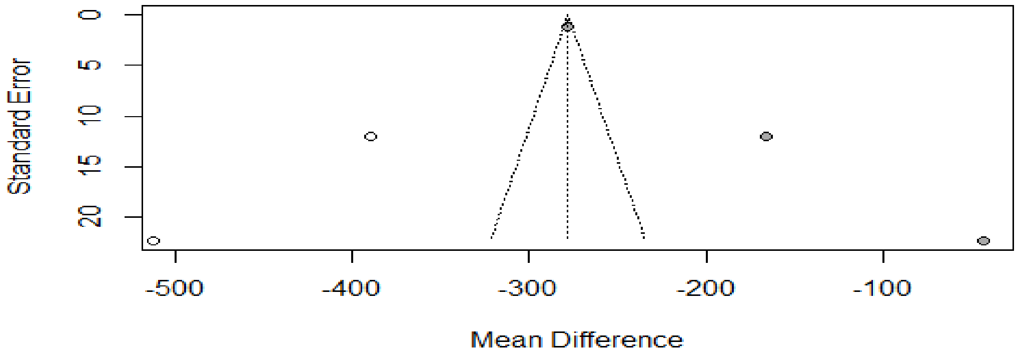

Figure 6: Funnel plots for publication bias in the studies selected for the analysis of the mean difference UIC of preeclamptic women and normotensive counterparts. The 2 imputed studies are represented by empty circles.

For peer review only

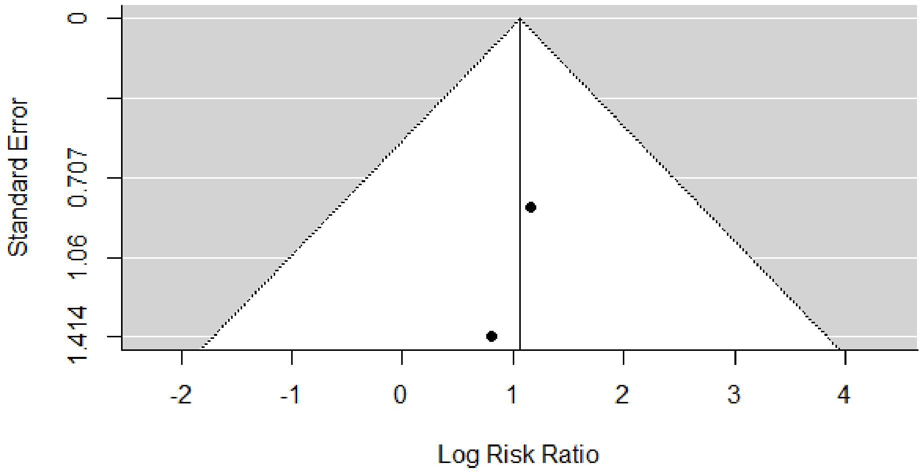

Figure 7: Funnel plot for the studies selected for the analysis of the risk of preeclampsia among women with <150 μg/L

PRISMA 2009 Checklist

Section/topic	#	Checklist item	Reported on page #
**TITLE**			
Title	1	Identify the report as a systematic review, meta-analysis, or both.	1
**ABSTRACT**			
Structured summary	2	Provide a structured summary including, as applicable: background; objectives; data sources; study eligibility criteria, participants, and interventions; study appraisal and synthesis methods; results; limitations; conclusions and implications of key findings; systematic review registration number.	2
**INTRODUCTION**			
Rationale	3	Describe the rationale for the review in the context of what is already known.	4
Objectives	4	Provide an explicit statement of questions being addressed with reference to participants, interventions, comparisons, outcomes, and study design (PICOS).	4
**METHODS**			
Protocol and registration	5	Indicate if a review protocol exists, if and where it can be accessed (e.g., Web address), and, if available, provide registration information including registration number.	4
Eligibility criteria	6	Specify study characteristics (e.g., PICOS, length of follow-up) and report characteristics (e.g., years considered, language, publication status) used as criteria for eligibility, giving rationale.	4
Information sources	7	Describe all information sources (e.g., databases with dates of coverage, contact with study authors to identify additional studies) in the search and date last searched.	5
Search	8	Present full electronic search strategy for at least one database, including any limits used, such that it could be repeated.	5
Study selection	9	State the process for selecting studies (i.e., screening, eligibility, included in systematic review, and, if applicable, included in the meta-analysis).	6
Data collection process	10	Describe method of data extraction from reports (e.g., piloted forms, independently, in duplicate) and any processes for obtaining and confirming data from investigators.	6
Data items	11	List and define all variables for which data were sought (e.g., PICOS, funding sources) and any assumptions and simplifications made.	6
Risk of bias in individual studies	12	Describe methods used for assessing risk of bias of individual studies (including specification of whether this was done at the study or outcome level), and how this information is to be used in any data synthesis.	6
Summary measures	13	State the principal summary measures (e.g., risk ratio, difference in means).	6
Synthesis of results	14	Describe the methods of handling data and combining results of studies, if done, including measures of consistency (e.g., I^2) for each meta-analysis.	6

For peer review only

PRISMA 2009 Checklist

Section/topic	#	Checklist item	Reported on page #
Risk of bias across studies	15	Specify any assessment of risk of bias that may affect the cumulative evidence (e.g., publication bias, selective reporting within studies).	6
Additional analyses	16	Describe methods of additional analyses (e.g., sensitivity or subgroup analyses, meta-regression), if done, indicating which were pre-specified.	6
**RESULTS**			
Study selection	17	Give numbers of studies screened, assessed for eligibility, and included in the review, with reasons for exclusions at each stage, ideally with a flow diagram.	7
Study characteristics	18	For each study, present characteristics for which data were extracted (e.g., study size, PICOS, follow-up period) and provide the citations.	7
Risk of bias within studies	19	Present data on risk of bias of each study and, if available, any outcome level assessment (see item 12).	7
Results of individual studies	20	For all outcomes considered (benefits or harms), present, for each study: (a) simple summary data for each intervention group (b) effect estimates and confidence intervals, ideally with a forest plot.	7
Synthesis of results	21	Present results of each meta-analysis done, including confidence intervals and measures of consistency.	7
Risk of bias across studies	22	Present results of any assessment of risk of bias across studies (see Item 15).	8
Additional analysis	23	Give results of additional analyses, if done (e.g., sensitivity or subgroup analyses, meta-regression [see Item 16]).	
**DISCUSSION**			
Summary of evidence	24	Summarize the main findings including the strength of evidence for each main outcome; consider their relevance to key groups (e.g., healthcare providers, users, and policy makers).	8
Limitations	25	Discuss limitations at study and outcome level (e.g., risk of bias), and at review-level (e.g., incomplete retrieval of identified research, reporting bias).	9
Conclusions	26	Provide a general interpretation of the results in the context of other evidence, and implications for future research.	9
**FUNDING**			
Funding	27	Describe sources of funding for the systematic review and other support (e.g., supply of data); role of funders for the systematic review.	9

From: Moher D, Liberati A, Tetzlaff J, Altman DG, The PRISMA Group (2009). Preferred Reporting Items for Systematic Reviews and Meta-Analyses: The PRISMA Statement. PLoS Med 6(7): e1000097. doi:10.1371/journal.pmed1000097

For more information, visit: **www.prisma-statement.org**.

For peer review only - http://bmjopen.bmj.com/site/about/guidelines.xhtml
